# RPA2 winged-helix domain facilitates UNG-mediated removal of uracil from ssDNA; implications for repair of mutagenic uracil at the replication fork

**DOI:** 10.1093/nar/gkab195

**Published:** 2021-03-30

**Authors:** Bodil Kavli, Tobias S Iveland, Edith Buchinger, Lars Hagen, Nina B Liabakk, Per A Aas, Tobias S Obermann, Finn L Aachmann, Geir Slupphaug

**Affiliations:** Department of Clinical and Molecular Medicine, NTNU Norwegian University of Science and Technology, NO-7491 Trondheim, Norway; Clinic of Laboratory Medicine, St. Olavs Hospital, Trondheim University Hospital, NO-7006 Trondheim, Norway; Department of Clinical and Molecular Medicine, NTNU Norwegian University of Science and Technology, NO-7491 Trondheim, Norway; Cancer Clinic, St. Olavs Hospital, Trondheim University Hospital, NO-7006 Trondheim, Norway; NOBIPOL, Department of Biotechnology and Food Science, NTNU Norwegian University of Science and Technology, N-7034 Trondheim, Norway; Department of Clinical and Molecular Medicine, NTNU Norwegian University of Science and Technology, NO-7491 Trondheim, Norway; Clinic of Laboratory Medicine, St. Olavs Hospital, Trondheim University Hospital, NO-7006 Trondheim, Norway; PROMEC Proteomics and Modomics Experimental Core at NTNU and the Central Norway Regional Health Authority, NO-7491 Trondheim, Norway; Department of Clinical and Molecular Medicine, NTNU Norwegian University of Science and Technology, NO-7491 Trondheim, Norway; Clinic of Laboratory Medicine, St. Olavs Hospital, Trondheim University Hospital, NO-7006 Trondheim, Norway; Department of Clinical and Molecular Medicine, NTNU Norwegian University of Science and Technology, NO-7491 Trondheim, Norway; Clinic of Laboratory Medicine, St. Olavs Hospital, Trondheim University Hospital, NO-7006 Trondheim, Norway; Department of Clinical and Molecular Medicine, NTNU Norwegian University of Science and Technology, NO-7491 Trondheim, Norway; Clinic of Laboratory Medicine, St. Olavs Hospital, Trondheim University Hospital, NO-7006 Trondheim, Norway; NOBIPOL, Department of Biotechnology and Food Science, NTNU Norwegian University of Science and Technology, N-7034 Trondheim, Norway; Department of Clinical and Molecular Medicine, NTNU Norwegian University of Science and Technology, NO-7491 Trondheim, Norway; Clinic of Laboratory Medicine, St. Olavs Hospital, Trondheim University Hospital, NO-7006 Trondheim, Norway; PROMEC Proteomics and Modomics Experimental Core at NTNU and the Central Norway Regional Health Authority, NO-7491 Trondheim, Norway

## Abstract

Uracil occurs at replication forks via misincorporation of deoxyuridine monophosphate (dUMP) or via deamination of existing cytosines, which occurs 2–3 orders of magnitude faster in ssDNA than in dsDNA and is 100% miscoding. Tethering of UNG2 to proliferating cell nuclear antigen (PCNA) allows rapid post-replicative removal of misincorporated uracil, but potential ‘pre-replicative’ removal of deaminated cytosines in ssDNA has been questioned since this could mediate mutagenic translesion synthesis and induction of double-strand breaks. Here, we demonstrate that uracil-DNA glycosylase (UNG), but not SMUG1 efficiently excises uracil from replication protein A (RPA)-coated ssDNA and that this depends on functional interaction between the flexible winged-helix (WH) domain of RPA2 and the N-terminal RPA-binding helix in UNG. This functional interaction is promoted by mono-ubiquitination and diminished by cell-cycle regulated phosphorylations on UNG. Six other human proteins bind the RPA2-WH domain, all of which are involved in DNA repair and replication fork remodelling. Based on this and the recent discovery of the AP site crosslinking protein HMCES, we propose an integrated model in which templated repair of uracil and potentially other mutagenic base lesions in ssDNA at the replication fork, is orchestrated by RPA. The UNG:RPA2-WH interaction may also play a role in adaptive immunity by promoting efficient excision of AID-induced uracils in transcribed immunoglobulin loci.

## Graphical Abstract

**Graphical Abstract:**
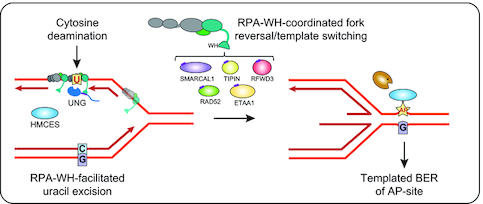
The WH-domain of RPA facilitates efficient UNG-mediated excision from RPA-coated ssDNA and may orchestrate error-free templated repair of the resultant AP-site.

## INTRODUCTION

Recent research indicates that DNA replication and deamination of cytosine and 5-methylcytosine (5-mC) are the major sources of cancer-associated mutations (1,2). This is supported by analysis of mutational signatures associated with C>T transition across a wide spectrum of human cancers (3). Two of the C>T signatures are correlated with age (‘clock-like’), further supporting a link to replication (4) (SBS1 and SBS5, https://cancer.sanger.ac.uk/cosmic/signatures/SBS/index.tt). Whereas C>T mutations in CpG contexts (SBS1) apparently originate from deamination of 5-mC, the biological processes underlying C>T transitions outside CpG sites (SBS5) remain obscure. One potential source of these C>T transitions is deamination of cytosine to uracil within single-stranded DNA (ssDNA) regions at the replication fork. Spontaneous and enzymatic cytosine deamination occurs 2–3 orders of magnitude faster in ssDNA than in dsDNA (5–7) and unless corrected before encounter of replicative polymerases, these would lead to C>T mutations after two replicative cycles. ssDNA regions continuously form at the lagging strand and could be extensive at either strand when the replicative polymerases pause or stall and uncoupled fork progression occurs (8).

**Figure 1.**
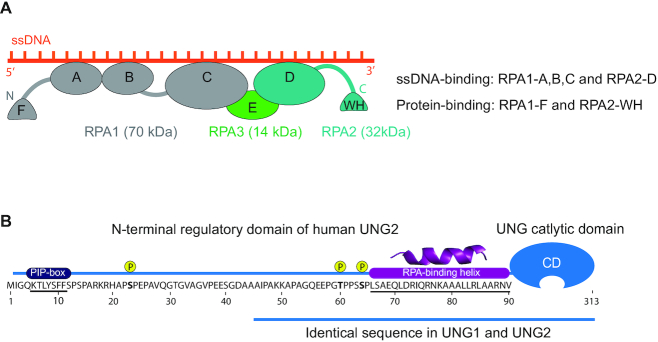
Protein domain architecture and important motifs in human RPA and UNG. (**A**) Domain structure and orientation of the RPA trimer (RPA1, RPA2, RPA3) bound to ssDNA. DNA-binding (RPA1-A,B,C; RPA2-D) and protein-binding (RPA1-F; RPA2-WH) domains are indicated. (**B**) Sequence and important motifs in the ∼90 aa N-terminal regulatory domain of UNG2. Binding motifs for PCNA (PIP-box), RPA and cell-cycle regulated phosphorylation sites are indicated. The UNG1 isoforms also contain residues 45–313, including the RPA-binding helix.

Once formed, ssDNA is rapidly coated with replication protein A (RPA), which is the major ssDNA-binding protein in eukaryotic cells and is essential for DNA replication, recombination and repair. In addition to stabilising ssDNA by preventing reannealing, secondary structures and digestion, RPA is an early responder to DNA damage and replication stress by constituting a binding platform for many proteins involved in genome maintenance (9,10), including the major uracil-DNA glycosylase UNG2 (11–13). These multifaceted functions are facilitated by the flexible structure of RPA. The heterotrimer is composed of the RPA1 (70 kDa), RPA2 (32 kDa) and RPA3 (14 kDa) subunits and contains four highly dynamic DNA-binding domains (DBDs) located within RPA1 (A, B, C) and RPA2 (D) as well as two flexible protein-binding domains; the N-terminal domain of RPA1 (F) and the RPA2 C-terminal winged-helix (WH) domain (Figure 1A). The trimer binds ssDNA with a defined 5′ to 3′ polarity and several binding modes have been proposed depending on ssDNA length and conformation, and the concentration of DNA, RPA and salts (14,15). In most previous models, primarily based on studies of partial RPA constructs, DBD A and B have been viewed as high affinity ssDNA binders, and DBD C and D as low-affinity binders. However, recent cryoEM, single-molecule and hydrogen-deuterium mass spectrometry (HDX-MS) studies have challenged this and rather indicate that the trimerization core (C, D and E) (Figure 1A) serves as the main anchor to ssDNA and that DBD A and B are more dynamic (16–19). In addition, RPA dynamics is apparently modulated by phosphorylation, primarily occurring at the N-terminus of RPA2 (17,20,21), and proteins binding to the flexible F and WH domains may promote internal rearrangement of the DBDs (19). The majority of the ∼40 identified RPA-interacting factors bind to the RPA1 subunit (N-terminal or DNA-binding domains), while a subset of seven proteins has been shown to interact with the WH domain of RPA2 (10) (Supplementary Figure S1). These WH-binding proteins are all central to genome maintenance processes, including replication fork regression and remodelling (SMARCAL1, TIPIN, and ETAA1), homology directed repair (HDR) (RAD52), nucleotide excision repair (XPA) and base excision repair (UNG) (11,22–26). In addition, the ubiquitin E3 ligase RFWD3 promotes replication checkpoint control, homologous recombination and fork restart (27–29). RPA-bound ssDNA has been shown to constitute a common intermediate in all these processes, most recently in a long patch BER sub-pathway in which RPA cooperates with RECQ1 to create a flap 5′ to the cleaved AP site (30). Although UNG efficiently excises uracil from ssDNA, at least *in vitro*, all downstream steps in BER require the dsDNA conformation to allow templated repair and to avoid formation of double strand breaks at the replication fork. Recent work from the laboratory of James T. Stivers demonstrated that RPA bound to ssDNA overhangs of junction DNA substrates mediated preferred targeting of UNG2 to uracil sites in the dsDNA region close to the junctions (31). This was likely facilitated by binding of UNG2 to RPA2 on the ssDNA overhangs. The flexible linker (∼30 aa), attaching the WH domain to the RPA2 domain D (Figure 1A), would position UNG2 in an optimal position to attack uracil sites in dsDNA near the junction (<21 bp). Moreover, they showed that the N-terminal region of UNG also binds to 5′-overhangs in absence of RPA and mediated excision bias for uracils in dsDNA close to the junction (<10 bp). Based on this they suggested that RPA-dependent and RPA-independent targeting to ssDNA may have a role at the replication fork in the removal of uracil that arise from dUTP incorporation during replication (U:A in dsDNA). Their assumption was based on an experimental model that measured uracil removal only from the dsDNA regions of the junction substrates. However, the steric hindrance imposed by the CMG helicase-complex or the PCNA–POL complex was not taken into consideration and such a mechanism would not prevent C>T transitions originating from cytosine deamination in replicative ssDNA. Removal of misincorporated uracil is more likely facilitated by the other major replicative hub protein PCNA, which binds the UNG2 PIP-box motif (12,32), encircles duplex DNA and recruits UNG2 to excise newly misincorporated uracil immediately behind the moving replication fork (13).

UNG is a multifunctional enzyme located both in the nucleus and mitochondria where it initiates error-free BER of misincorporated uracils from U:A base pairs as well as deaminated cytosines from U:G mispairs. In addition, it acts as a regulator of HIV-1 infectivity (33) and has been implicated in TET-mediated DNA demethylation (34). Interestingly, both UNG and RPA are required for somatic hypermutation (SHM) and class switch recombination (CSR) where they induce mutagenic processing of uracils generated by activation-induced cytidine deaminase (AID)-mediated deamination of cytosines in immunoglobulin (Ig) loci (35). The multiple functions of UNG are apparently regulated by its flexible N-terminal domain (31,36–42). UNG is expressed as two major isotypes, generally referred to as nuclear UNG2 and mitochondrial UNG1. These isoforms differ only in their N-terminal sequences, which guide the enzymes to the nucleus and mitochondria, respectively (Figure 1B). This traditional view was recently challenged when we identified a novel nuclear UNG1 isotype variant that performed efficient uracil removal in the nuclear genome, as well as supported processing of AID-generated uracil and induction of CSR (32). Nuclear UNG2 is the only isoform that contains a PCNA-binding motif (PIP-box) (Figure 1B), which likely targets the enzyme to perform immediate post-replicative removal of newly incorporated uracil (U:A) from the nascent strands (13). Although the same study suggested that a region in UNG2 overlapping the PIP-box could bind RPA *in vitro*, subsequent analyses of UNG2 mutants demonstrated that this region did not contribute to RPA binding by intact UNG2 (12,32). Interestingly, UNG2 isotype-specific knockout cells, expressing only UNG1 isoforms, revealed that PCNA binding is neither important for efficient repair of misincorporated uracil nor for the role of UNG in adaptive immunity (32). In addition to the UNG2-specific PIP-box, all identified UNG isoforms contain the RPA-binding helix motif located adjacent to the C-terminal catalytic domain (Figure 1B) (11,12,32,43), but the biological function of this interaction is still not understood.

UNG has a high preference for excision of uracil from ssDNA compared to dsDNA (36). However, *in vivo*, ssDNA is bound by RPA and other ssDNA-binding proteins that protect the DNA strand against attack by nucleases and other DNA-modifying enzymes. We previously reported that uracil excision by mitochondrial UNG1 from ssDNA is strongly inhibited by the human mitochondrial ssDNA-binding protein mtSSB (44). A potential function of this could be to delay uracil removal from replicative single-stranded mtDNA until the dsDNA conformation is restored. In the same study, we also found that RPA mediated virtually no inhibition of UNG1 activity. To what degree RPA hinders access of UNG2 to uracil in RPA-coated ssDNA, has previously not been investigated. Earlier studies in our group demonstrated that RPA mediated a moderate reduction of UNG2-mediated uracil excision from dsDNA substrates, whereas excision from ssDNA was moderately enhanced (12,45). However, these studies were undertaken with large molar excess of long DNA substrates (nick-translated calf thymus DNA) and without pre-incubation of RPA/DNA.

Here, we explored the functional relevance of the interaction between RPA and UNG by employing uracil-containing oligonucleotides preincubated with large molar excess RPA to ensure pre-formation of RPA/DNA complexes. This would be biologically relevant, since the number of RPA molecules within human cells is two orders of magnitude higher than UNG as well as the single-strand selective uracil DNA glycosylase SMUG1 (46). We demonstrate that UNG2 mediates highly efficient uracil excision from RPA-bound ssDNA, while the same substrate is protected against attack from the uracil-DNA glycosylase SMUG1. We further show that this ability of UNG depends on the specific interaction between the RPA2-WH domain and the N-terminal RPA-binding helix motif in UNG. Moreover, we show that phosphorylation and ubiquitination of the UNG N-terminal domain regulate the RPA2-WH interaction and uracil excision from RPA-coated ssDNA.

We propose a model in which the RPA2-WH domain promotes uracil excision of deaminated cytosines in ssDNA at the replication fork and coordinates fork remodelling to restore dsDNA and allow downstream error-free BER. RPA is also detected together with RNA polymerase II in transcribed regions of active genes (47), where it can function as a sensor of R-loops (48). In activated B-cells, this mediates recruitment of AID to immunoglobulin genes to facilitate SHM and CSR (49,50). It is thus tempting to speculate that RPA located at actively transcribed *Ig* loci recruits UNG to ssDNA in R-loops to promote mutagenic processing of AID-generated uracil during adaptive immunity in B cells.

## MATERIALS AND METHODS

### Recombinant proteins and mutagenesis

Plasmid encoding human trimeric RPA (p11d-tRPA) was a gift from Prof. Marc S. Wold (University of Iowa). Recombinant RPA was expressed in *Escherichia coli* BL21(DE3) RIPL and purified with Affi-Gel Blue (BioRad), hydroxyapatite (BioRad), and Mono Q (GE Healthcare) chromatography as described (51). A plasmid expressing RPA lacking the WH domain (RPA-ΔWH, codon 190 of RPA2 mutated to a TGA stop codon) was generated by the Q5 site-directed mutagenesis kit (New England Biolabs) according to manufacturer's instructions. Cloning of the RPA2-WH domain (RPA2 residues 172–270) into the pTYB12 expression vector was performed by a sequence- and ligation-independent strategy as described (52). RPA2-WH-intein fusion protein was expressed in ZYP-5052 autoinduction medium at 16°C overnight and the WH domain purified according to standard protocol (52). Recombinant human UNG2 and SMUG1 were prepared as described previously (36,45,53). Constructs expressing N-terminally deleted and mutated UNG proteins were generated by the Q5 site-directed mutagenesis kit and Quick-change site-directed mutagenesis kit (Stratagene), respectively. Mutations were verified by Sanger-sequencing (GATC Biotech AG, Germany) and confirmed by mass spectrometry (MS) analysis of purified proteins. Cloning, expression, and purification of the ^15^N-labelled UNG2 N-terminal (residues 1–93) was as described previously (52).

### Uracil excision assays with RPA-coated and naked DNA substrates

3′-FAM-labelled, PAGE-purified oligonucleotide substrates were from Sigma-Aldrich. Unless otherwise indicated, the substrate (25 nt) harboured uracil at position 10 in a polyC sequence to avoid secondary structures (U10-25*: CCACCCCCC**U**CCCCCCCCCCCCCCC-FAM). Double-stranded substrate was generated by annealing (heating followed by slow cooling) U10-25* to a non-labelled complementary oligo (A16-25: GGGGGGGGGGTGGGG**A**GGGGGGTGG). All assays were performed at 22°C in 10 mM Tris–HCl pH 7.5, 50 mM NaCl, 1 mM DTT, 0.1 mM EDTA, 0.5 mg/ml BSA.

By monitoring the activity of the catalytic UNG domain (0.2 nM UNG-CD), lacking the N-terminal RPA-binding helix), we found that >400 nM RPA fully abolished uracil excision when pre-incubated with 100 nM U10-25* ssDNA substrate (data not shown), indicating that at such conditions all ssDNA was bound to RPA and not accessible for processing by UNG-CD. Based on this, 10-fold molar excess of RPA over substrate was employed in subsequent experiments unless otherwise stated. DNA substrate (100 nM final) and RPA (1 μM final) were mixed and incubated on ice for 15 min to form RPA/ssDNA complexes. Varying amounts of UNG were then added, and the mixtures incubated for 10 min in a water bath. To avoid unspecific interactions and binding of enzyme and DNA to the assay tube surface, low-DNA binding tubes and excess of BSA was used in all reactions. Reactions were quenched and AP sites cleaved in 10% piperidine at 90°C for 20 min. Samples were dried by vacuum centrifugation and suspended in formamide-containing loading buffer. Product and substrate were separated in 12% PAGE/7M urea–0.5× TBE gels, bands visualised in ChemiDoc™ Imager (Bio-Rad) and quantified by Image Lab software (Bio-Rad). Importantly, all assays in which two parameters were compared (e.g. RPA-coated versus naked substrate or WT versus mutated protein) were performed in parallel with the exact same DNA/protein dilutions.

### MicroScale Thermophoresis (MST)

Recombinant human RPA trimer was labelled and purified using the Monolith NT™ Protein Labelling RED-MALEIMIDE kit (NanoTemper Technologies, Germany) according to the manufacturer's protocol. UNG peptides were from Proteogenix (Schiltigheim, France). MST was performed on Monolith NT.115 (NanoTemper Technologies) using standard capillaries with settings 60% MST and 50% LED power in optimised MST buffer (50 mM Tris–HCl pH 8, 150 mM NaCl, 10 mM MgCl_2_, 0.05% Tween-20, 0.5 mg/ml BSA). A constant amount of RPA (330 nM) and a concentration gradient (∼10 nM–200 μM) of each peptide were used in all experiments. *K*_d_ values were calculated from four runs for each experiment using the MO-Affinity Analysis software (NanoTemper Technologies).

### RPA affinity capture

Peptides (EV-34, pEV-34 and ppEV-34) were covalently coupled to epoxy beads (Dynabeads M-270 Epoxy, Thermo Fisher) as described by the producer. Coupled beads (15 μl) were added to HeLa whole cell extract (WCE, 1 mg protein) and incubated for 30 min before washing in PBS and elution in LDS loading buffer. Input and affinity captured RPA were quantified by western analysis using monoclonal rabbit anti-RPA2 [EPR2877Y] (ab76420) primary antibody (1:1000, Abcam) and swine anti-rabbit HRP (1:5000, Dako) as secondary antibody.

### Circular dichroism

All CD-experiments were performed on Chirascan (Applied Photophysics) using a 1 mm cuvette at 25°C. Samples were measured within the range of 180–260 nm in 20 mM phosphate buffer pH 7.0, 10 mM NaCl. The measured millidegree from all spectra were transformed to mean residue ellipticity θ. The 222/208 nm ratios were used to compare the helicity of the peptides.

### NMR and paramagnetic relaxation enhancement analysis

The purified WH domain was MTSL-labelled at its single cysteine residue (C219) by adding 10-fold molar excess of MTSL (*S*-[(1-oxyl-2,2,5,5-tetramethyl-2,5-dihydro-1H-pyrrol-3-yl)methyl] methanesulfonothioate) dissolved in DMSO to a sample of RPA2-WH in 20 mM phosphate buffer pH 7.0, 10 mM NaCl. The mixture was incubated in darkness overnight to complete the reaction. MTSL labelled RPA2-WH was extensively washed with 20 mM phosphate buffer pH 7.0, 10 mM NaCl. NMR spectra of ^15^N-labelled N-terminal UNG2 in presence of 1.5 molar excess of MTSL-RPA2-WH before and after addition of ascorbic acid were recorded at 25°C in NMR buffer (20 mM phosphate buffer pH 7.0, 10 mM NaCl, H_2_O/D_2_O 9:1) on a Bruker Ascend 800 MHz Avance III HD NMR spectrometer equipped with 5 mm *z*-gradient TXI (H/C/N) cryogenic probe and processed with Bruker TopSpin version 3.2/3.5. Spectral analysis and peak intensities were determined by using CARA version 1.9.1.7.

### *In vitro* ubiquitination

*In vitro* ubiquitination for E2 ligase determination was performed on purified recombinant human UNG2 using ubiquitination kit from Enzo Life Sciences (BML-UW9920-0001) according to the manufacturer's instructions, using 0.036 μg/μl His-UNG2 and HeLa protein extract (0.6 μg/ml final) as E3 ligase donor. Ubiquitination of UNG2 Lys to Arg mutants were performed by the same protocol using UBCH2 as E2 ligase. Ubiquitinated UNG for uracil-excision assays was generated with UBCH2 in absence of E3 ligase by using 0.068 μg/μl N-terminally deleted UNG2 and 0.05 μg/μl BSA instead of HeLa protein extract. Mock samples were treated identically, except that ATP was not included in the reaction. Ubiquitination was verified by western blot analysis using polyclonal rabbit anti-UNG (PU59, made in-house) primary antibody and swine anti-rabbit HRP (Dako) as secondary antibody and single ubiquitination at K78 was identified by LC–MS/MS analysis.

### UNG affinity capture by Ugi

The UNG-specific inhibitor protein Ugi (54) was covalently coupled to epoxy beads (Dynabeads M-270 Epoxy, Thermo Fisher) as described by the producer. Coupled beads (15 ul) were added to 1 mg HeLa WCE and incubated for 30 min before washing in PBS. Affinity captured proteins were trypsinised directly on the beads for MS analysis.

### Mass spectrometry analysis

Proteins were digested and desalted as described (55,56) evaporated to dryness and resuspended in 0.1% formic acid prior to analysis on a LC–MS/MS platform consisting of an Easy-nLC 1000 UHPLC interfaced with an LTQ-Orbitrap Elite hybrid mass spectrometer via a nanospray ESI ion source (Thermo Scientific/Proxeon). Peptides were injected onto a C-18 trap column (Acclaim PepMap100 (75 μm i. d. × 2 cm, C18, 5 μm, 100 Å, Thermo Scientific) and separated on a C-18 analytical column (Acclaim PepMap100 (75 μm i.d. × 50 cm, C18, 3 μm, 100 Å, Thermo Scientific) using a 84 min gradient from 10 to 40% CH_3_CN, 0.1% formic acid at a flow rate of 250 nl/min. Peptides were analysed in positive ion- and data dependent acquisition (DDA) mode using the following parameters: Electrospray voltage 1.9 kV, CID fragmentation with normalised collision energy 35, automatic gain control (AGC) target value of 1E6 for Orbitrap MS and 1E3 for MS/MS scans. Each MS scan (*m*/*z* 400–1600) was acquired at a resolution of 120 000 FWHM, followed by 20 MS/MS scans triggered for intensities above 500, at a maximum ion injection time of 200 ms for MS and 50 ms for MS/MS scans.

## RESULTS

### UNG2 efficiently excises uracil from RPA-coated ssDNA

**Figure 2.**
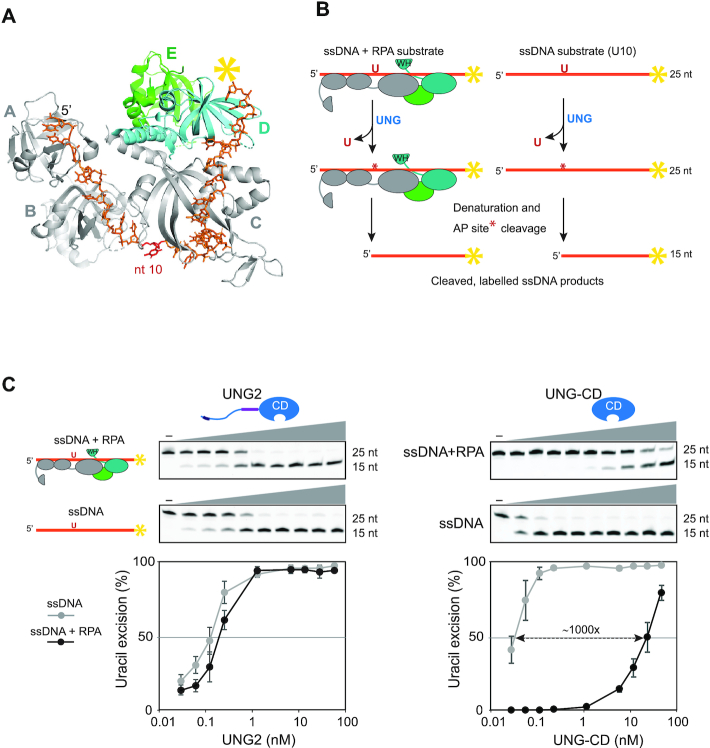
UNG2 promotes highly efficient uracil excision from RPA-coated ssDNA. (**A**) 3D structure of the RPA heterotrimer bound to ssDNA (25 nt). The DNA binding domains (A, B and C) in RPA1 (grey) and D in RPA2 (cyan) are indicated. RPA3 is green and the direction of the DNA strand and nt position 10 are indicated. The structure is visualised using PyMOL2 software with PDB accession code 4GNX (*Ustilago maydis)* (57). (**B**) Illustration of the uracil-excision assay used to analyse UDG activity. The yellow asterisk indicates 6FAM labelling at the 3′ end of the uracil-containing ssDNA substrate (25 nt) and product (15 nt). (**C**) Uracil-excision activity with RPA (1 μM)-coated and naked ssDNA substrates (100 nM U10-25*) for full-length UNG2 and N-terminally truncated UNG (UNG-CD). Upper panels show representative PAGE gels for one experiment. Each curve represents mean activity calculated from three independent experiments, (*I*_15nt_/*I*_15nt+25nt_) × 100%. Standard deviations are indicated with error bars. Note that the UNG concentrations (x-axes) are represented on a logarithmic scale.

UNG2-mediated excision of uracil from dsDNA was recently shown to be stimulated by the presence of an RPA-coated ssDNA junction (31). However, whether UNG2 can access uracil embedded within RPA-coated ssDNA itself has not been investigated. To address this, we employed a 25 nt oligonucleotide substrate harbouring uracil at position 10 and 6FAM-label at the 3′-end. This construct was based on the crystal structure of *Ustilago maydis* RPA trimer complexed to a 25 nt ssDNA (57) (Figure 2A). The substrate was pre-incubated with RPA to form a stable complex in which the RPA trimer would cover the entire DNA strand. After pre-incubation in the presence/absence of RPA, the substrates were incubated with UNG2 or UNG-CD as control, and uracil excision quantified as described (Figure 2B). As illustrated in Figure 2C, UNG2 was almost equally capable of excising uracil from the RPA-coated and naked substrates. By contrast, the highly efficient UNG-CD displayed 1000-fold reduced activity with RPA-coated ssDNA compared to naked DNA substrate.

To investigate whether positioning of uracil or length of ssDNA substrate have impact on the results, we prepared a 25 nt oligonucleotide identical to U10-25*, but with uracil shifted three nucleotides in the 3′ direction (U10U13-25*). Based on the RPA:ssDNA complex structure (Figure 2A), this position is predicted to be less accessible. We also made extended versions (43 nt) of both DNA substrates, harbouring 18 additional 3′-terminal nucleotides (U10–43* and U13–43*) (Supplementary Figure S2A). Uracil excision from the three modified substrates (100 nM) by UNG-CD was completely abolished when coated with 1 μM RPA (Supplementary Figure S2B). Conversely, full-length UNG2 was able to excise uracil from all substrates even at 4 μM RPA, but with varying efficiency. The substrate-dependent variation observed and the ability of UNG2 to excise uracil with only slightly reduced efficiency (U10 substrates) in presence of 10 000-fold molar excess of RPA compared to UNG (40-fold compared to DNA) demonstrates that UNG is not titrated out by free RPA molecules that are not bound to DNA.

In the 25 nt substrates, shifting uracil in the 3′ direction mediated markedly reduction in excision (Supplementary Figure S2C, left panel), suggesting increased steric hindrance by DBD-C, as predicted (Figure 2A). U10 in the 43 nt substrate was excised as efficiently as in the 25 nt substrate. Notably, in the longer substrate, much less reduced excision was observed when uracil was shifted to the 13 position, compared to the 25 nt substrate. This may be contributed by a more ‘relaxed’ positioning of RPA along the length of the substrate. Disregarding the number of RPA complexes bound to the 43 nt substrate, U13 would then be closer to DBD-A/B than in the 25 nt substrate since the 5′ half is identical in both substrates and alternative binding must occur towards the 3′ half. This would be in agreement with the findings that DBD-A and B are more dynamic than the trimerization core (C, D and E) (16–18), thereby allowing increased access to U13 in the longer substrate.

In separate experiments we compared UNG2 and the single-strand selective monofunctional uracil DNA glycosylase SMUG1 using the U13–43* ssDNA substrate. SMUG1 is catalytically slow compared to UNG (58), prefers double-stranded substrates (59) and is expressed at 3–10-fold lower levels across human cell lines than UNG (46,53). Whereas UNG2 peaks during S-phase (32,45,60), SMUG1 is constitutively expressed through the cell cycle and, in contrast to UNG2, is not localized to replication foci (58,60). Although this suggests that SMUG1 does not have an important function in uracil sanitation at replication forks, a potential function in ssDNA outside of replication forks has not been investigated. As shown in Supplementary Figure S3, SMUG1 activity was virtually blocked by RPA-coating of the ssDNA substrate. These experiments demonstrate that (i) access to uracil in ssDNA is restricted by binding of RPA to the substrate, (ii) UNG2, but not SMUG1, can excise uracils from RPA-bound ssDNA and (iii) the N-terminal regulatory domain of UNG2 promotes access to catalytic removal of these uracils.

### Access to uracil in RPA-coated ssDNA depends on specific interaction between the WH domain and the UNG N-terminal helix

**Figure 3.**
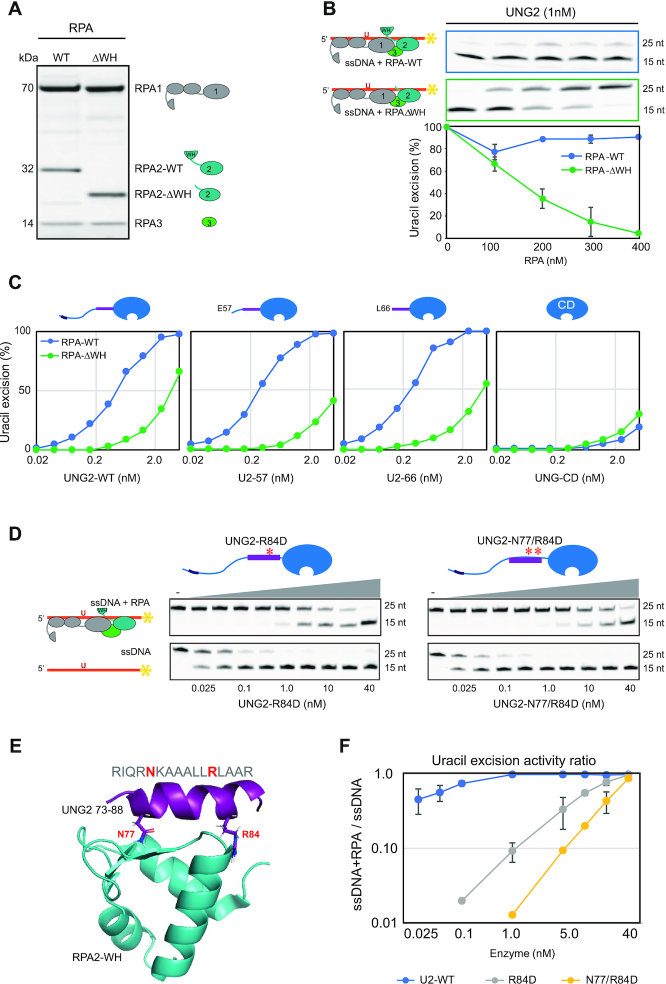
Uracil excision from RPA-coated ssDNA depends on the C-terminal RPA2-WH domain and the UNG2 N-terminal RPA-binding helix. (**A**) Coomassie blue-stained SDS-PAGE gel of purified RPA trimer (1 μg) containing either RPA2-WT or RPA2-ΔWH. (**B**) RPA-ΔWH, but not RPA-WT, inhibits uracil-excision from ssDNA by UNG2. Upper panels show representative PAGE gels for one experiment. Substrate (25 nt) and product (15 nt) bands are indicated. 100 nM ssDNA substrate and 0–400 nM RPA (WT or ΔWH) were used in each reaction. Curves represent mean activity calculated from three independent experiments. Standard deviations are indicated as error bars. (**C**) UNG mutants with partial N-terminal truncation, but that still contain the RPA-binding helix, retain activity on RPA(WT)-coated ssDNA. RPA (500 nM, WT or ΔWH) and 100 nM ssDNA were used in the assays. Note logarithmic scale on the x-axes. Mutations of UNG2 residues involved in RPA binding disrupt the ability to target uracil in RPA-coated ssDNA (D–F). (**D**) PAGE gels showing uracil-excision experiments with RPA-coated ssDNA (1 μM RPA and 100 nM ssDNA) and naked ssDNA (100 nM) together with UNG2 single mutant (R84D) and double mutant (N77/R84D). (**E**) NMR structure of a peptide segment (UNG2 residues 73–88) bound to the RPA2-WH domain. Original side chains of the mutated UNG residues are indicated. The figure was generated using PyMOL2 and PDB coordinates 1DPU. (**F**) Calculated uracil excision activity ratios from several experiments as shown in panel D, representing activity with RPA-coated ssDNA (1 μM RPA and 100 nM ssDNA) divided by activity with 100 nM naked ssDNA (ssDNA+RPA/ssDNA). Each curve represents the mean of three independent experiments and standard deviations are indicated as error bars.

To investigate whether the ability of UNG2 to excise uracil from the RPA-coated substrate was dependent on the WH domain of RPA2, we deleted the domain and purified the corresponding RPA trimer (RPA-ΔWH) (Figure 3A). Next, we compared UNG2 uracil-excision activity with ssDNA substrate preincubated with increasing concentrations of either RPA-WT or RPA-ΔWH. Notably, deletion of the WH domain resulted in a markedly reduced capability of UNG2 to excise uracil from the RPA-coated substrate (Figure 3B). Whereas uracil excision was essentially eliminated in the presence of 400 nM RPA-ΔWH, there was no decrease in uracil excision by RPA-WT. Thus, the ability of UNG2 to target uracil in RPA-coated ssDNA is facilitated by its interaction with the RPA2-WH domain.

To further identify which parts of the ∼90 aa N-terminal domain of UNG2 that contribute to substrate recognition in RPA-coated ssDNA, we generated partial UNG2 N-terminal deletion mutants starting at either aa 57 (U2–57) or 66 (U2–66). In contrast to UNG-CD (starting at UNG2 aa 93) (Figure 1B), U2–57 and U2–66 that both contain the helix motif known to bind RPA2-WH, excised uracil from RPA-WT-coated substrate as effectively as full-length UNG2 (Figure 3C). Conversely, when the ssDNA substrate was bound to RPA lacking the WH domain, the uracil excision activities were low for all four UNG forms (Figure 3C). This demonstrates that the UNG2 N-terminal helix (starting at residue 66) and the RPA2-WH domain are both necessary for uracil excision from RPA-coated ssDNA.

Finally, we mutated UNG2 N-terminal helix residues N77 and R84, which are directly involved in RPA2-WH binding and abolish interaction with RPA when mutated to aspartate (12) (Figure 3D,E). Whereas full-length UNG2-WT excised uracil from naked and RPA-coated substrates with comparable efficiency (Figure 2C), both mutants displayed compromised excision from the RPA-coated substrate (Figure 3D). This effect was highly significant when activity ratios between the two substrates where calculated from several independent experiments in which RPA-coated and naked substrates were analysed in parallel with the same enzyme dilutions (Figure 3F). In summary, this demonstrates that UNG has a unique capability among the human UDGs to excise uracil from RPA-bound ssDNA and that this depends on the specific interaction between the UNG N-terminal helix and the RPA2-WH domain.

### Cell-cycle regulated phosphorylations adjacent to the UNG2 N-terminal helix regulate binding to RPA

**Figure 4.**
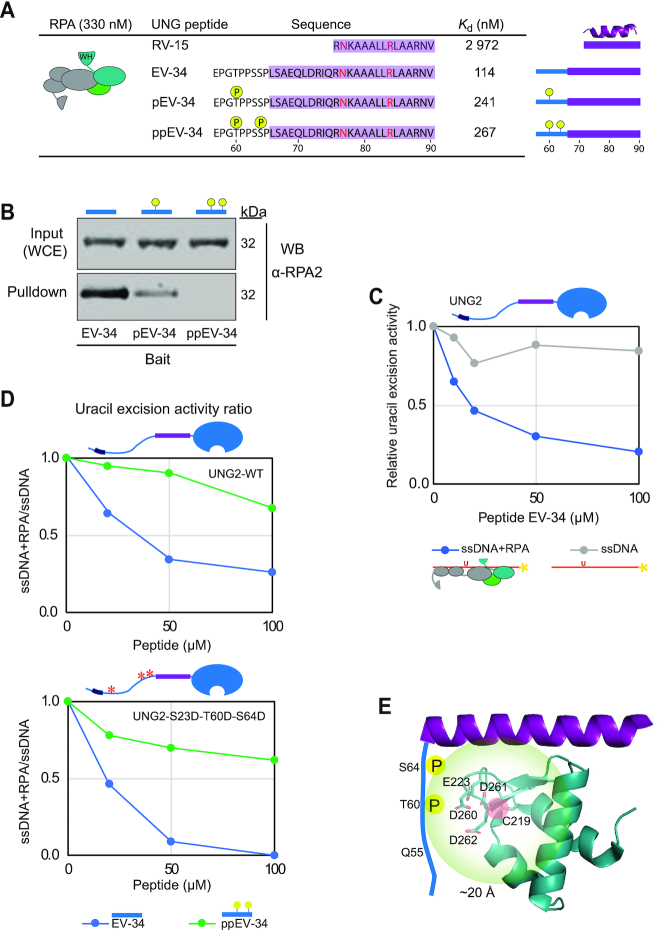
UNG phosphorylation regulates RPA binding affinity. (**A**) RPA binding affinity to various UNG peptides. Dissociation constants (*K*_d_) were measured by MicroScale Thermophoresis (MST). Residues forming the UNG RPA-binding helix, N77 and R84 essential for RPA binding, and adjacent phosphorylated sites are highlighted. (**B**) Western blot showing pull-down of endogenously expressed RPA from HeLa whole cell extract (WCE) using the non-, mono- (pT60), and di-phosphorylated (pT60, pS64) UNG peptide-coated beads as bait. (**C**) Peptide competition assay showing reduced access of UNG2 specifically to uracil in RPA-coated ssDNA in the presence of EV-34 peptide. Curves represent the mean activity measured in two independent experiments using 0.4 nM UNG2, 1 μM RPA and 100 nM ssDNA (U10–25*) substrate. (**D**) Peptide competition experiments on UNG2-WT and UNG2 P-mimicking mutant, with the same conditions as in panel C, comparing non-phosphorylated (EV-34) and di-phosphorylated (ppEV-34) peptides. The curves represent normalised uracil-excision activity ratios (RPA+ssDNA/ssDNA). (**E**) Structural interpretation of the results, including paramagnetic relaxation enhancement analysis of RPA2-WH domain (MTSL-labelled at C219) and N-terminal UNG2 residues 1-93 (^15^N-labelled). The structural model illustrates that the UNG2 Q55-S63 region is within 20 Å from RPA2 C219 residue. The negatively charged patch on the surface of the RPA2-WH domain consists of side chains E223, D260, D261 and D262. The structural model is visualised using PyMOL2 software based on the PDB coordinates 4MQV.

Protein-protein interactions are commonly regulated by post-translational modifications (PTMs). We previously identified two stepwise and cell-cycle regulated phosphorylations in the UNG2 N-terminal domain just upstream of the RPA-binding helix (Figure 1B), and phospho-mimicking mutants suggested that these regulate affinity towards RPA (45). To investigate this further, we synthesised a panel of peptides, including the short RPA2-WH-binding core peptide (RV15, UNG2 residues R76-V90) (43) and three N-terminally extended versions thereof containing 3, 10 and 19 additional residues, respectively (Supplementary Figure S4). The longest, EV-34 (UNG2 residues E57-V90), harbours both phosphorylation sites and was synthesised as non-phosphorylated (EV-34), mono-phosphorylated on T60 (pEV-34) and di-phosphorylated on T60 and S64 (ppEV-34) peptides (Figure 4A). Secondary structures were determined by CD spectroscopy and secondary chemical shift data from NMR (Supplementary Figure S4). Neither RV-15 nor RV-18 displayed any stable secondary structure in solution while LV-25 folded as a full helical peptide, in accordance with a previous study (61). All EV-34 peptides displayed helical fold for the same ∼25 aa (UNG2 residues 66–90), while the ∼9 aa N-terminal part was non-structured (UNG2 residues 57–65). Notably, phosphorylation did not cause any apparent change in peptide helicity (Supplementary Figure S4).

To investigate whether phosphorylation at T60 and S64 regulates RPA-binding, we first measured the RPA binding affinity for the EV-34 peptides by MST, using increasing concentrations of non-labelled peptides and a constant amount of RPA trimer labelled with NT-67 RED dye. The identified dissociation constants (*K*_d_) show that phosphorylation at T60 mediated two-fold reduction in RPA binding whereas double phosphorylation at T60 and S64 resulted in a further decrease (Figure 4A). The analyses also revealed that the EV-34 peptides displayed 10–26-fold stronger binding to RPA than the short RV-15 peptide. This demonstrates that UNG2 residues outside the ‘core’ (RV-15) contribute to the interaction surface with RPA.

Next, we covalently attached the EV-34 peptides to magnetic epoxy beads to investigate the effect of T60 and S64 phosphorylations on RPA pull-down from HeLa whole cell extracts. These experiments confirmed that the single- and double phosphorylations have an increasingly negative impact on RPA binding (Figure 4B). In another approach, we tested whether the peptides could outcompete binding of UNG2 to RPA and thereby inhibit uracil excision from RPA-coated ssDNA. In the presence of increasing concentrations of EV-34, UNG2 activity was reduced (Figure 4C). In accordance with the increased *K*_d_ in MST assay and reduced pull-down efficiency of RPA, phosphorylated ppEV-34 peptides showed less inhibition of the uracil excision activity than unphosphorylated EV-34 (Figure 4D, upper panel). The inhibitory effect of the EV-34 peptide was even more pronounced for a UNG2 phospho-mimicking mutant with decreased affinity to RPA compared to WT-UNG2 (45) (Figure 4D, lower panel).

We previously reported chemical shift assignment of full-length UNG2 (52) (BRMB entry 27133). For a structural assessment of how phosphorylation of T60 and S64 regulates RPA binding, we used paramagnetic relaxation enhancement (PRE) NMR measurements to probe intermolecular interactions. The RPA2-WH domain contains a single solvent-exposed cysteine (RPA2 C219), making it an excellent candidate for attaching a PRE label like MTSL to it. To measure PREs, NMR spectra of the ^15^N-labelled N-terminal region of UNG2 were recorded in the absence and presence of the MTSL-labelled RPA2-WH domain. Using this technique, NMR signals of residues near (within 20 Å) to the PRE label will experience a reduction in signal intensity, as a function of residence time and distance to the label (62). While none of the residues in the helical part of the UNG2 N-terminal region were affected, signals of residues in the regions Q55-S63 were markedly reduced (Supplementary Figure S5). This suggests that the region in UNG2 encompassing the two phosphorylation sites (T60 and S64) is located close (<20 Å) to the RPA2 C219 residue. Near this residue, there is a negatively charged surface patch consisting of side chains from E223, D260, D261 and D262. It is likely that, upon phosphorylation of UNG2 T60 and S64, there is electrostatic repulsion between the phosphate groups and the negatively charged patch on the surface of RPA2, leading to a decrease in the interaction strength (Figure 4E). Thus, cell cycle-dependent phosphorylation of UNG2 that reduces binding to the RPA2-WH domain can regulate access of UNG2 to uracil in RPA-coated ssDNA.

### Ubiquitination at K78 in the UNG2 N-terminal helix stimulates uracil-excision from RPA-coated ssDNA

The UNG2 protein level and phosphorylation status are tightly regulated through cell cycle. T60 and S64 phosphoforms gradually accumulate through S-phase, preceding a mono-ubiquitinated isoform that accumulates in G2 (45). To identify the ubiquitination site, we synchronised HeLa cells with double thymidine block and harvested cells in G2. UNG isoforms were then enriched from the G2 cell extract, using magnetic beads coupled to the UNG-inhibitor protein Ugi. Mass spectrometry analysis identified a single ubiquitination site harbouring Gly-Gly at K78 (Supplementary Figure S6A). This Ub site has also been reported in several high-throughput screens (www.phosphosite.org), but the ubiquitin ligases involved and the functional consequences of K78 ubiquitination remain unknown.

**Figure 5.**
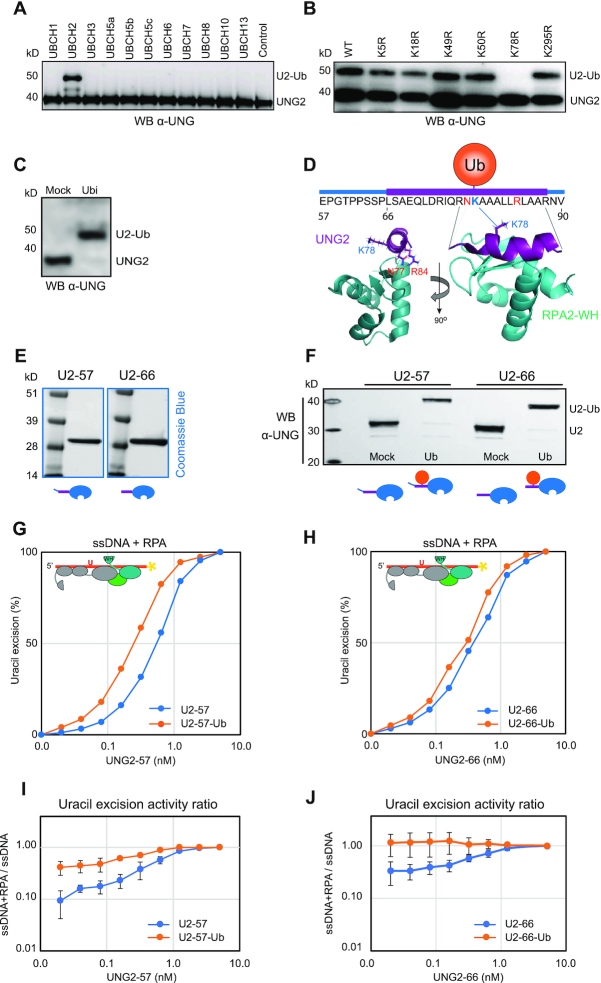
Ubiquitination at K78 in the UNG RPA-binding helix stimulates uracil-excision from RPA-coated ssDNA. (**A**) Western blot demonstrating *in vitro* ubiquitination of purified recombinant human UNG2 by a panel of different E2 ligases with HeLa nuclear extract as E3 ligase donor. (**B**) UBCH2-mediated *in vitro* ubiquitination of purified recombinant UNG2 Lys to Arg (K to R) mutants verified by western analysis. HeLa nuclear extract was added to the reactions. (**C**) Western blot showing near complete UBCH2-mediated *in vitro* ubiquitination of purified recombinant UNG2-WT in absence of E3 ligase. (**D**) Position of K78 within the helix and position/direction of the side chain in the complex viewed from two angles (UNG peptide:RPA2-WH, PDB:1DPU). (**E**) Coomassie blue-stained SDS-PAGE gels of purified recombinant UNG2 N-terminal deletion mutants starting at residue 57 (U2-57) and residue 66 (U2-66), respectively. (**F**) Western blot demonstrating *in vitro* ubiquitination of UNG2 deletion mutants U2-57 and U2-66. (**G**) Uracil excision assay employing either ubiquitinated or mock-treated UNG2 deletion mutant U2-57 with RPA-coated ssDNA substrate (500 nM RPA and 100 nM U10–25* ssDNA). The curves represent mean values from three independent experiments. (**H**) Similar experiment as in G using the mock-treated and ubiquitinated forms of UNG2 deletion mutant U2-66. Curves represent mean values from four independent experiments. Results from experiments with naked ssDNA (run in parallel) are illustrated in Supplementary Figure S7. (**I**) Uracil excision ratio (activity with RPA-coated ssDNA substrate divided by activity with naked ssDNA) for ubiquitinated (U2-57-Ub) and mock-treated (U2–57) UNG deletion mutant. Curves represent mean values of three experiments performed in parallel with RPA-coated and naked U10-25* ssDNA. (**J**) Similar experiments as in Figure I performed with the ubiquitinated (U2–66-Ub) and mock-treated (U2-66) UNG deletion mutant (four experiments). Standard deviations are indicated as error bars (I, J).

To identify potential ubiquitin ligases that target UNG2, we first subjected recombinant UNG2 to *in vitro* ubiquitination, using a panel of 11 E2 ubiquitin ligases and HeLa nuclear extract as E3 donor. UNG2 was readily and uniquely mono-ubiquitinated by the UBCH2 E2 ligase (Figure 5A). Moreover, in accordance with the endogenous Ub site identified in G2-enriched cells, a screen of UNG2 single mutants (all K sites individually mutated to R) confirmed that UBCH2 in presence of the E3 ligase source uniquely ubiquitinates K78 *in vitro* (Figure 5B). UBCH2 has previously been shown to work as an E3-independent E2 ligase for histone H2A (63). To test if UBCH2 could perform E3-independent ubiquitination of UNG2, we replaced HeLa nuclear extract with BSA. Surprisingly, this increased the ubiquitination efficiency to almost 100% (Figure 5C), compared to the partial ubiquitination obtained in presence of E3 ligase donor (Figure 5A and B). However, the increased ubiquitination efficiency came with reduced specificity, as MS analysis also revealed partial ubiquitination at K5 and K50 in the N-terminal domain in the absence of E3 ligase (data not shown).

K78 is strongly conserved and is positioned within the RPA-binding helix (Supplementary Figure S6B). Structural inspection shows that the side chain extrudes from the helix on the opposite side of the WH-binding UNG residues N77 and R84 (Figure 5D), suggesting that ubiquitinated UNG may still interact with RPA. However, the size of ubiquitin (76 aa) is comparable to the UNG2 N-terminal domain and when situated in the N-terminal helix it may influence RPA binding, as suggested (32). To address this, we subjected the purified (Figure 5E) UNG2 N-terminal deletion mutants U2-57 and U2–66 (to avoid K5/K50 ubiquitination) to E3-independent *in vitro* ubiquitination as above. We obtained near 100% ubiquitination of K78 in both deletion mutants (Figure 5F), and MS analysis revealed no additional ubiquitination sites (data not shown). We first compared the capability of fully K78-ubiquitinated versus mock-ubiquitinated (reactions lacking ATP) forms of both mutants to excise uracil from naked ssDNA. This revealed no (U2–57) or modestly decreased (U2–66) uracil excision by the ubiquitinated enzymes (Supplementary Figure S7). Conversely, both ubiquitinated enzymes displayed modestly increased activity with RPA-coated ssDNA substrates compared to the corresponding mock treated non-modified enzymes (Figure 5G and H). The UNG enzymes were analysed with both RPA-coated and naked DNA substrates in parallel and significantly increased activity against RPA-coated substrates by K78 ubiquitination was demonstrated when comparing the activity ratios (ssDNA+RPA/ssDNA) calculated from several independent experiments (Figure 5I and J). This demonstrates that ubiquitination of the UNG RPA-binding helix does not block RPA binding but rather modestly stimulates the capability of UNG2 to excise uracil from RPA-coated ssDNA.

Finally, we investigated to what degree pre-binding of UNG to RPA affected UBCH2-mediated ubiquitination of K78. Here, we found that RPA did not reduce ubiquitination of UNG2 (or U2–66), and as expected, no ubiquitination occurred within the catalytic domain (Supplementary Figure S8). Thus, ubiquitination of K78 occurs on both unbound and RPA-bound UNG2 and may be a means to both promote recruitment and to increase the binding strength of UNG2 already bound to RPA2-WH.

### RPA stimulates uracil excision from dsDNA by substrate binding and WH-mediated UNG recruitment

It has been shown that RPA can bind and transiently unwind double-stranded DNA (64,65) and stimulate uracil excision, likely by creating single-stranded substrate (66). To address the role of the WH domain in this context, we generated a dsDNA substrate (A:U10-25*) with an A:U base pair in position 10 and high GC content to stabilise the double-helix structure (*T*_m_ = 88°C). We first investigated whether binding of the WH domain to the N-terminal helix allosterically activated UNG, by analysing activity of U2–66 in the presence of excess purified WH domain. As shown in Supplementary Figure S9A, addition of the free WH domain did not affect uracil excision from dsDNA neither in presence nor absence of RPA, demonstrating that the WH domain does not stimulate uracil excision by allosteric activation of UNG. Next, we monitored activity of a fixed amount of UNG2 or N-terminally truncated versions, in the presence of increasing amounts of purified WH domain. Whereas the free WH domain had little effect on uracil excision from naked ss- and dsDNA substrates, uracil excision was markedly reduced from both RPA-bound substrates (Supplementary Figure S9B). This indicates that the WH domain must be present as part of the RPA complex to promote uracil excision from RPA-bound ss- and dsDNA and that outcompeting this interaction by free WH domain markedly decreases excision.

**Figure 6.**
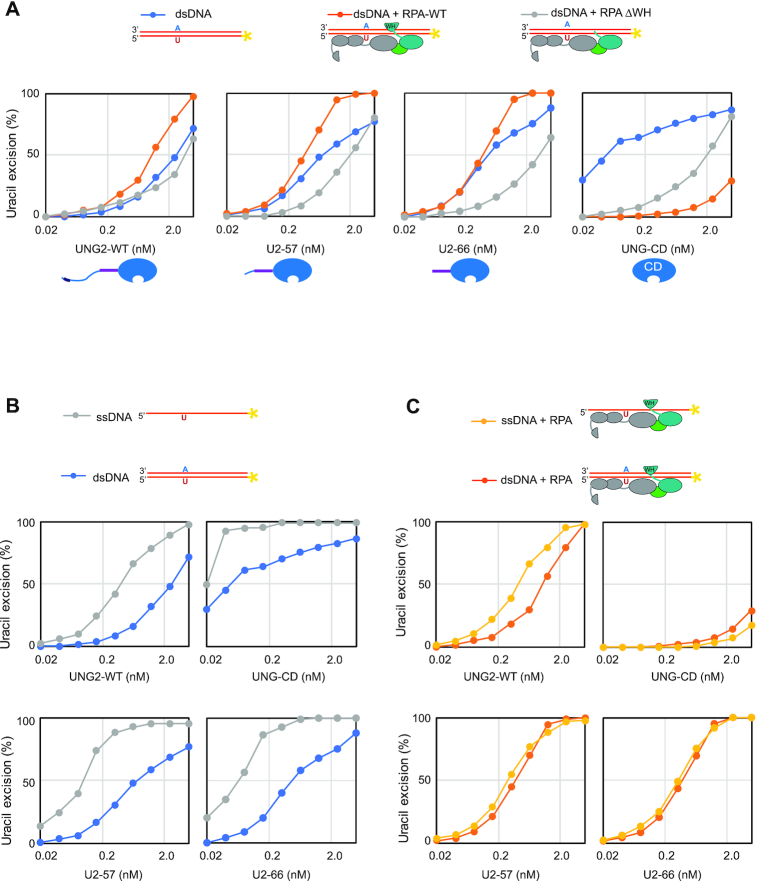
RPA stimulates uracil excision from dsDNA by DNA binding and RPA2-WH domain mediated UNG2 recruitment. Uracil excision assay with UNG2-WT, N-terminally truncated UNG variants U2-57 and U2-66, and the UNG catalytic domain (UNG-CD). 100 nM U10–25* DNA substrate (ss or ds) were used in all experiments. 500 nM RPA (WT or ΔWH) was preincubated with the substrate where indicated. (**A**) Uracil excision experiments analysing the effect of RPA-WT and RPA-ΔWH together with dsDNA substrate. (**B**) Experiments comparing naked ssDNA and dsDNA uracil-excision activity. (**C**) Experiments comparing ssDNA and dsDNA, both preincubated with RPA.

To further investigate the mechanism whereby RPA stimulates uracil excision from dsDNA, we pre-incubated the dsDNA substrate with/without RPA-WT or RPA-ΔWH prior to addition of UNG2 or N-terminally truncated versions thereof (Figure 6A). UNG2, U2-57 and U2-66 were all stimulated by RPA-WT but not by RPA-ΔWH, whereas UNG-CD was strongly inhibited by both RPA variants and required ∼50- and 200-fold increased enzyme concentrations to convert similar amounts of substrate in presence of RPA-ΔWH and RPA-WT, respectively (Figure 6A, right panel). This supports that RPA stimulates excision of uracil from dsDNA by a mechanism dependent on both the RPA2-WH domain and the UNG N-terminal helix, as previously suggested (31). In the above experiments we also included the corresponding ssDNA substrates in parallel as controls. In accordance with previous analyses, UNG2 and N-terminally truncated variants displayed higher uracil-excision with ssDNA than with dsDNA (Figure 6B). By contrast, the activity profiles with ssDNA and dsDNA substrates in presence of RPA were almost overlapping (Figure 6C). These results conform with a model in which RPA targets UNG2 to uracil in both ssDNA and dsDNA, thereby promoting uracil excision from both substrates with similar efficiency *in vivo*. Finally, we analysed to what extent K78 ubiquitination (U2-66) affected uracil excision from dsDNA substrates in the presence or absence of RPA. For direct comparison, these experiments were run in parallel with ssDNA substrates. A weak reduction of uracil excision was observed from naked ssDNA for the ubiquitinated form, whereas a modest increase was observed from the corresponding dsDNA substrate (Supplementary Figure S10A). These effects were essentially abolished when the substrates were preincubated with either WT RPA or RPA lacking the WH domain, and the activity curve profiles became virtually identical for ssDNA and dsDNA (Supplementary Figure S10 B and C, respectively), supporting that RPA converts the dsDNA substrates into ssDNA (64–66).

## DISCUSSION

**Figure 7.**
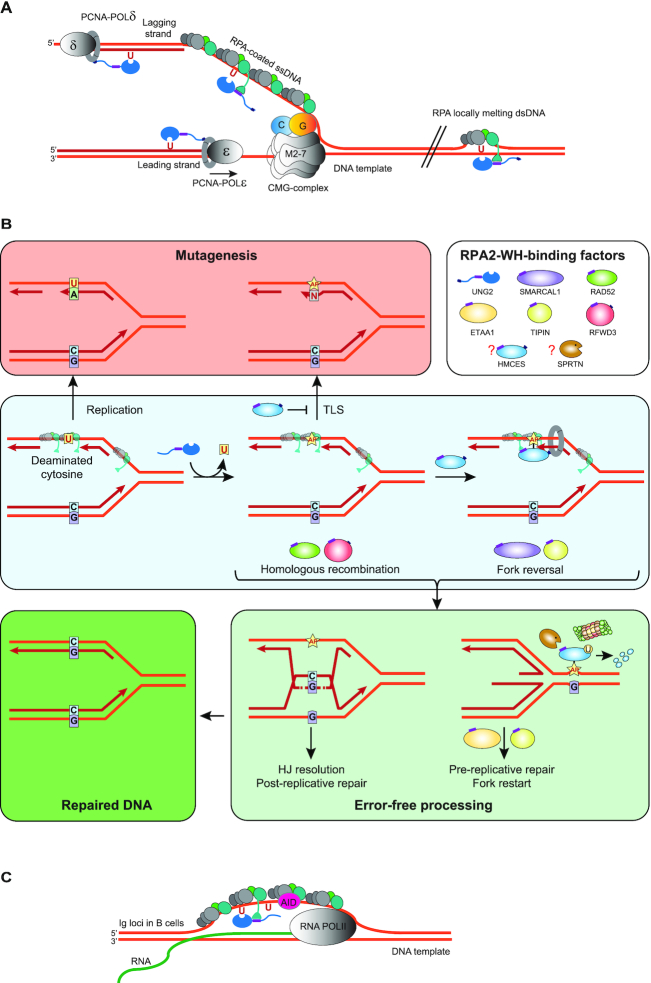
Model showing targeting of UNG to ssDNA regions in replication forks and transcription loops. (**A**) Recruitment of UNG2 to post-replicative U:A repair is facilitated by binding of the N-terminal PIP-box to PCNA (nascent strands in red). Correspondingly, recruitment of UNG2 to mutagenic, deaminated cytosines in ssDNA template in front of the replicative polymerases (illustrated in lagging strand only) is mediated by binding of the N-terminal helix to the flexible WH domain of RPA2. Targeting to RPA-bound ssDNA in locally melted dsDNA outside of replication forks is also indicated. CMG complex; replicative helicase complex (Cdc45/Mcm2–7/GINS). (**B**) Hypothetical model illustrating repair of uracil generated by cytosine deamination in replicative ssDNA. White box illustrates known and suspect (HMCES) RPA2-WH -binding proteins. During unperturbed replication, the majority of replicative ssDNA is formed at the lagging strand, which would face the highest risk of cytosine deamination. If not removed prior to encounter by POLD, this would be 100% mutagenic. Similarly, uracil excision from the ssDNA template and fill-in by TLS polymerases would be highly error-prone (red box). These mutagenic events are counteracted by UNG2, which excises the uracil, and by HMCES, which crosslinks to the AP site and blocks TLS. Blocked replication induces RPA2-WH -dependent recruitment of SMARCAL1, which promotes fork reversal and migration of the AP site into dsDNA ahead of the fork (light green box, right). Prior to further processing, crosslinked HMCES is degraded by the DNA-structure specific protease SPRTN or by proteasomal degradation, thereby facilitating error-free pre-replicative BER. Alternatively, RPA2-WH recruits RAD52 to induce template switching, allowing dGMP insertion across the AP site by employing the nascent leading strand as template (light green box, left). HJ resolution would then allow post-replicative BER. (**C**) RPA-mediated targeting of AID and UNG to ssDNA regions at transcription sites (e.g. variable and switch regions of *Ig* loci in B cells).

By interacting with the N-terminal helix of UNG, the RPA2-WH domain promotes efficient uracil-excision from RPA-coated ssDNA. At replication forks, this would be biologically relevant to avoid mutations due to cytosine deamination in the ssDNA regions preceding the replicative polymerases (Figure 7A). Binding of RAD52 to the RPA2-WH domain was recently proposed to induce loading of RAD52 towards the 3′-end of a 30 nt oligonucleotide with concomitant reduced binding of DBD-D and other RPA elements towards the 3′-end (19). It is less likely that UNG can displace the RPA trimerization core to the same extent as RAD52, given its smaller binding interface with ssDNA (67) than the oligomeric RAD52 (PDB 5XRZ). Our initial results employing oligonucleotides of varying length and uracil positioning rather conform to a model in which the UNG:RPA2-WH interaction promotes internal rearrangement of the DBDs and increased accessibility to the region bound by DBD-A/B. However, the exact mechanism whereby the UNG:RPA2-WH interaction allows access to uracil must await structural studies involving UNG2 and the intact RPA trimer bound to ssDNA.

The increased accessibility to uracil in RPA-coated ssDNA observed after mono-ubiquitination of UNG2 K78 in the WH-binding helix was unexpected. Potentially, this modification fine-tunes binding to RPA2-WH by counteracting the weakened binding mediated by T60 and S64 phosphorylation. These phosphorylations occur in late S-G2 phase (45) and may facilitate release of UNG2 from RPA2 during replication fork disassembly. This would expose the largely unstructured N-terminal domain of UNG2, thereby inducing proteasomal degradation in late S-G2 in the absence of polyubiquitination. We recently demonstrated that histone deacetylase inhibitors mediated hyperacetylation of UNG2 and robust proteasome-dependent degradation, potentially mediated by K78 acetylation that would block ubiquitination (68). In agreement with this, Bao *et al.* (69) recently demonstrated that acetylation of K78 was a prerequisite for binding of the E3 ligase UHRF1. This mediated polyubiquitination of a yet unidentified lysine in the UNG2 N-terminal and proteasomal degradation of UNG2. Upon ROS exposure, UNG2 is deacetylated at K78 and this could be a means to increase the UNG2 protein level to sanitize oxidative base lesions (69). Since mono-ubiquitination would block acetylation of K78, it is reasonable to anticipate that this would also promote UNG2 stability. A small fraction of UNG2 persists through G2/M, among which a mono-ubiquitinated species dominates (45). It is possible that cells maintain a small amount of K78-ubiquitinated UNG2 through G2/M-phase to conduct specific tasks, potentially associated with CENP-A assembly (70,71) or processing of uracil in RPA-coated ssDNA arising from DNA catenates at centromeres/rDNA loci or late replication intermediates (72). Notably, the dsDNA-specific uracil–DNA glycosylase TDG is oppositely cell-cycle regulated compared to UNG2, and peaks in G2/M (73). K78-ubiquitination of UNG2 could thus be a means of functionally segregating these two glycosylases in G2/M by increasing association with RPA-coated ssDNA.

Our demonstration that the UNG:RPA2-WH interaction mediates a 1000-fold increased ability to excise uracil from RPA-coated ssDNA (Figure 2C) conforms with a model where PCNA and RPA target UNG to excise genomic uracil in dsDNA and ssDNA, respectively, including RPA-dependent targeting of UNG to deaminated cytosines in the lagging strand ss template (Figure 7A). This may also hold true in the leading strand when DNA polymerase ϵ is blocked. Many lesions on the leading strand template do not block the replicative CMG helicase, but pause the polymerase, potentially mediating uncoupling and formation of ssDNA in the leading strand template (74).

### A model for downstream processing of uracil in replicative ssDNA

AP sites generated from uracil excision cannot be further processed by AP endonuclease 1 (APE1, APEX1) when present in RPA-coated ssDNA (75), probably to safeguard against formation of double-strand breaks. Thus, to allow safe backbone cleavage and faithful BER, the dsDNA conformation must be restored prior to further processing of the AP site. This may be facilitated by fork reversal, which recently has emerged as a global response to replication arrest (76,77). AP sites are potent blocks of replicative polymerases but may be bypassed by error-prone translesion synthesis (TLS) (78,79). However, TLS may be counteracted by the newly discovered suicide enzyme 5-hydroxymethylcytosine (5hmC) binding, ES-cell-specific (HMCES). HMCES forms covalent crosslinks to AP sites in ssDNA (80–82) and was suggested to travel with replication forks bound to PCNA via a C-terminal PIP-box (80). Whereas the PIP-box is believed to recruit housekeeping proteins to the replication forks, the alternative APIM motif apparently mediate stress-induced recruitment of proteins to PCNA (83). Closer inspection of the proposed PCNA-binding motif in HMCES actually reveals that it conforms better with APIM (consensus: R/K- F/W/Y- L/I/V/A- L/I/V/A- K/R (84)) (Supplementary Figure S11) than with the PIP-box (consensus: QxxΨxxϑϑ, where Ψ is an aliphatic hydrophobic residue (L, M, I,V), ϑ is aromatic (most often Y or F), and x can be any amino acid). In support of this, HMCES-deficient cells are hypersensitive to DNA-damaging agents that induce AP sites (80). Very recently, HMCES was directly linked to processing of deaminated cytosines in ssDNA at replication forks. By fusing the ssDNA-specific cytidine deaminase APOBEC3A to a mutant estrogen receptor, Mehta *et al.* (85) induced nuclear localisation of APOBEC3A. This mediated reduced cell viability and slowed replication fork progression due to TLS polymerase engagement, both of which were exacerbated by inactivation of HMCES. Collectively, these studies strongly suggest that HMCES plays an important role in protecting cells from mutagenic and cytotoxic effects of uracil-mediated AP sites formed in ssDNA, but do not explain how the AP sites are further processed. We hypothesise that RPA contributes to orchestrate this through its RPA2-WH domain. Among the seven proteins known to bind the WH domain, UNG, HMCES, SMARCAL1, RFWD3 and TIPIN travel with replication forks, as demonstrated by iPOND coupled with mass spectrometry (80,86). Despite numerous efforts, we have not been able to delete the WH domain from RPA2 by CRISPR/Cas9-mediated genome-editing, supporting that this domain may be essential even in unperturbed cells. Based on our results and other studies, we propose a replication-dependent model (Figure 7B) in which the RPA2-WH domain coordinates a process involving uracil-induced replication fork arrest by recruitment of UNG to excise uracil in the ssDNA template and generate replication-blocking AP sites. The AP site is then crosslinked to HMCES, which may arrive bound to PCNA (80). Potentially, HMCES may also be recruited via RPA2 since it (annotated as C3orf37) was found to bind RPA2 with high confidence in three BioPlex human interactome studies (87–89). In support of this, the C-terminal of HMCES that contains the proposed PCNA-binding motif also contains an overlapping motif that is highly homologous to the RPA2-WH-binding motif of RFWD3 (Supplementary Figure S11), rendering an RPA-mediated ‘passing the baton’ mechanism (90) of the lesion from UNG to HMCES possible. Downstream processing of free and crosslinked AP sites may follow different paths mediated by RPA2-WH. Recruitment of SMARCAL1 would promote fork reversal to translocate the AP site into dsDNA and allow error-free pre-replicative BER. Here, RPA-mediated recruitment of RAD52 would hinder uncontrolled fork reversal and unscheduled degradation (91,92). Potentially, concomitant nascent strand synthesis by template switching may occur within the chicken-foot structure, aided by RAD52, which is involved in most aspects of HDR (25,93). After repair is complete, RPA2-WH co-ordinates the action of TIPIN and ETAA1 to facilitate replication restart. Alternatively, initial recruitment of RAD52 promotes correct insertion of dGMP across the AP site by employing the nascent leading strand as template. Here, RFWD3 could play an important role by ubiquitinating and removing both RPA and RAD51 from DNA damage sites to promote homologous recombination (29). Subsequent resolution of the recombination intermediate would then allow error-free post-replicative BER.

There are several details that remain to be elucidated to validate such a model. For example, it is not clear to what degree different WH-binding factors can be dynamically exchanged on a single RPA molecule during repair. Since multiple copies of RPA are bound to replicative ssDNA, damage processing may involve coordinated action of the WH-binding proteins at different RPA molecules. It is also possible that the RPA2-WH domain simply promotes repair by mediating elevated concentrations of the interacting proteins at the replication fork. However, our demonstration that the WH interaction directly facilitates access to uracil in RPA-bound ssDNA, suggests that downstream steps may also be coordinated by WH-binding. Various binding affinities (*K*_d_) of UNG2-derived peptides to the WH domain of RPA2 have been reported. Xie *et al.* (61) found *K*_d_ = 6.6 μM for a 24 aa peptide by isothermal calorimetry and Mer *et al.* (43) reported *K*_d_ < 1 μM for a 16 aa peptide by NMR titration. Values within this range have also been reported for binding of full-length UNG2 to RPA (39,66). Although the *K*_d_ values vary depending on the methods employed, values for the other WH-binding factors are also in the low micromolar to nanomolar range (43,61,94,95), suggesting transient and interchangeable binding. Furthermore, we find that binding of UNG to the WH domain can be decreased by phosphorylation and modestly increased by ubiquitination, indicating that binding is highly regulated. Several PTMs have been reported in the RPA2-WH domain as well as in regions flanking the RPA2-WH binding motifs (www.phosphosite.org) of its binding partners. SMARCAL1, which contains a binding motif highly homologous to UNG and TIPIN is phosphorylated at the upstream S2. This corresponds to a position between T60 and S64 in UNG2 and could thus contribute to lowering the affinity to RPA (Supplementary Figure S1A). Moreover, TIPIN is ubiquitinated at K207, which is situated at the same position as K78 in UNG and could increase affinity towards RPA (Supplementary Figure S11). RFWD3 is subject to either acetylation or ubiquitination of two lysins in the RPA2-WH binding motif (K364 and K370) and that could constitute affinity switches. The ETAA1 RPA2-WH binding motif contains a serine (S894) that has been reported to be phosphorylated in stressed and unstressed cells (www.phosphosite.org) and that conforms to phosphorylation both by cyclin-dependent kinases (SP) and Akt (RxRxxS/T). RAD52 is phosphorylated at S251 and acetylated at K262 and K274 in the binding motif. The potential roles of these modifications in orchestrating DNA repair remain, however, to be investigated.

It is also not known what fraction of AP sites in replication fork ssDNA that become crosslinked to HMCES prior to induction of fork regression or recombination, and to what degree crosslinked HMCES is completely degraded prior to further processing of the AP site. HMCES degradation was originally suggested to occur via ubiquitin-mediated proteasomal degradation (80). Very recently, a novel DNA-structure specific protease named SPRTN was reported (96). SPRTN contains two DNA-binding interfaces able to read out structural features and DNA context, thereby allowing controlled degradation of crosslinked proteins close to perturbations such as nicks, gaps and bubbles in dsDNA. It is tempting to speculate that a crosslinked AP site would similarly activate SPRTN protease activity and thus facilitate templated BER and fork restart.

Finally, some of the WH domain binding factors as well as several other proteins are known to bind RPA subunits outside of the RPA2-WH domain, including a number of DNA helicases and translocases (97,98). To what extent these factors contribute to repair of uracil and potentially other ssDNA base lesions such as oxidised bases (99) at the replication fork remains to be established.

UNG and RPA also have converging functional roles in adaptive immunity. These roles are likely independent of replication but rely on RPA and UNG processing of uracil lesions in ssDNA. In B-cells of vertebrates, AID deaminates cytosines in ssDNA of transcribed Ig loci (100). UNG-mediated removal of these cytosines from Ig variable (V) and switch (S)-regions is central to SHM and CSR, respectively (101–103). In this process, RPA binds to AID and stabilises the ssDNA regions to mediate deamination (49,50). Conceivably, RPA could then recruit UNG in the next step to mediate excision of the deaminated cytosines and induce error-prone processing (Figure 7C).

To conclude, we here demonstrate that the interaction between the RPA2-WH domain and the UNG N-terminal helix facilitates uracil excision from RPA-coated ssDNA. Moreover, we show that this interaction as well as its functional consequences are regulated by phosphorylation and ubiquitination in the UNG N-terminal region. The ability of UNG to excise uracil in RPA-coated ssDNA may be important to prevent mutagenic replication of deaminated cytosine by inducing replication arrest followed by fork reversal, repair, and replication restart. The flexible RPA2-WH domain may play a crucial role in orchestrating these events. During adaptive immunity the ability of UNG to excise uracil in RPA-coated ssDNA may, however, be important to facilitate mutagenic processing of AID-generated uracil in actively transcribed Ig genes. A potential role of RPA in the choice between repair or mutagenesis in the latter process remains to be investigated.

## Supplementary Material

gkab195_Supplemental_File
