# Supplementary material for: RPA2 winged-helix domain facilitates UNG-mediated removal of uracil from ssDNA; implications for repair of mutagenic uracil at the replication fork
Source: Nucleic Acids Res. 2021 Mar 30;49(7):3948–66. doi: 10.1093/nar/gkab195 (PMC8053108; doi:10.1093/nar/gkab195)
Supplement: gkab195_Supplemental_File [file gkab195_supplemental_file.pdf]

A

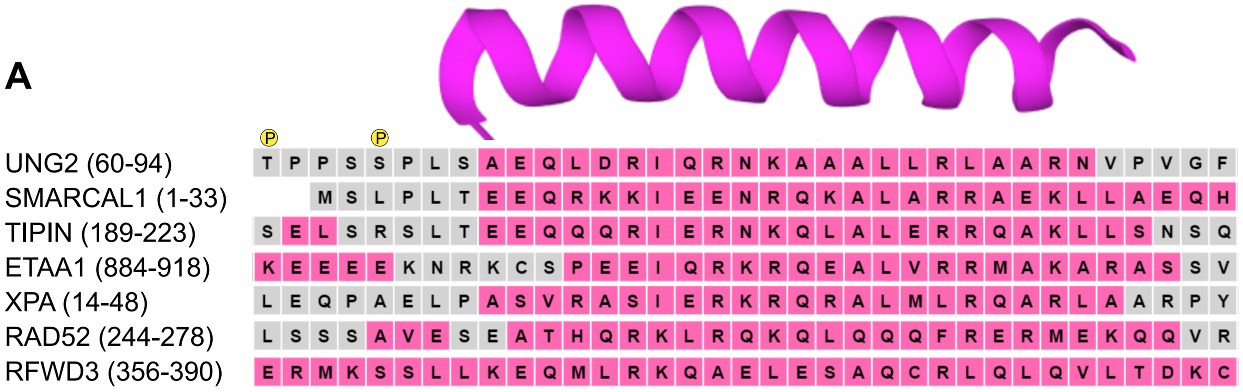

B

Protein	Function	RPA2-WH binding motif
UNG	BER, CSR, SHM	LSAEQLDRIQRNKAAALLRLAARNV
SMARCAL1	Fork reversal helicase	LTEEQRKKIEENRQKALARRAEKLL
TIPIN	Replication fork stabilizer	LTEEQQQRIERNKQLALERRQAKLL
ETAA1	Replication fork restart	NRKCSPEEIQRKRQEALVRRMAKAR
XPA	NER	LPASVRASIERKRQRALMLRQARLA
RAD52	Homologous recombination	ESEATHQRKLRQKQLQQQFRERMEK
RFWD3	E3 ligase FANCW	LLKEQMLRKQAELESAQCRLQLQVL

Supplementary Figure S1: A) Secondary structure prediction of sequences harbouring RPA2-WH binding motifs, with helical residues in pink (http://bioinf.cs.ucl.ac.uk/psipred/). The illustrated helix is from SMARCAL1 (PDB 4MQV). Cell-cycle regulated phosphorylations flanking the RPA-WH-binding helix of UNG2 are indicated (Hagen et al., EMBO J. 27, 2008, 51-61). **B)** Proposed RPA-WH-binding regions and protein functions, with amino acid colouring according to HeliQuest 34 (Gautier et al.,Bioinformatics 24, 2008:2101, https://heliquest.ipmc.cnrs.fr/).

A

U10-25*	CCA CCC CCC **U**CC CCC CCC CCC CCC C [6FAM]
U13-25*	CCA CCC CCC CCC **U**CC CCC CCC CCC C [6FAM]
U10-43*	CCA CCC CCC **U**CC CCC CCC CCC CCC CGC GTT TTT TTT TTT TGC G [6FAM]
U13-43*	CCA CCC CCC CCC **U**CC CCC CCC CCC CGC GTT TTT TTT TTT TGC G [6FAM]

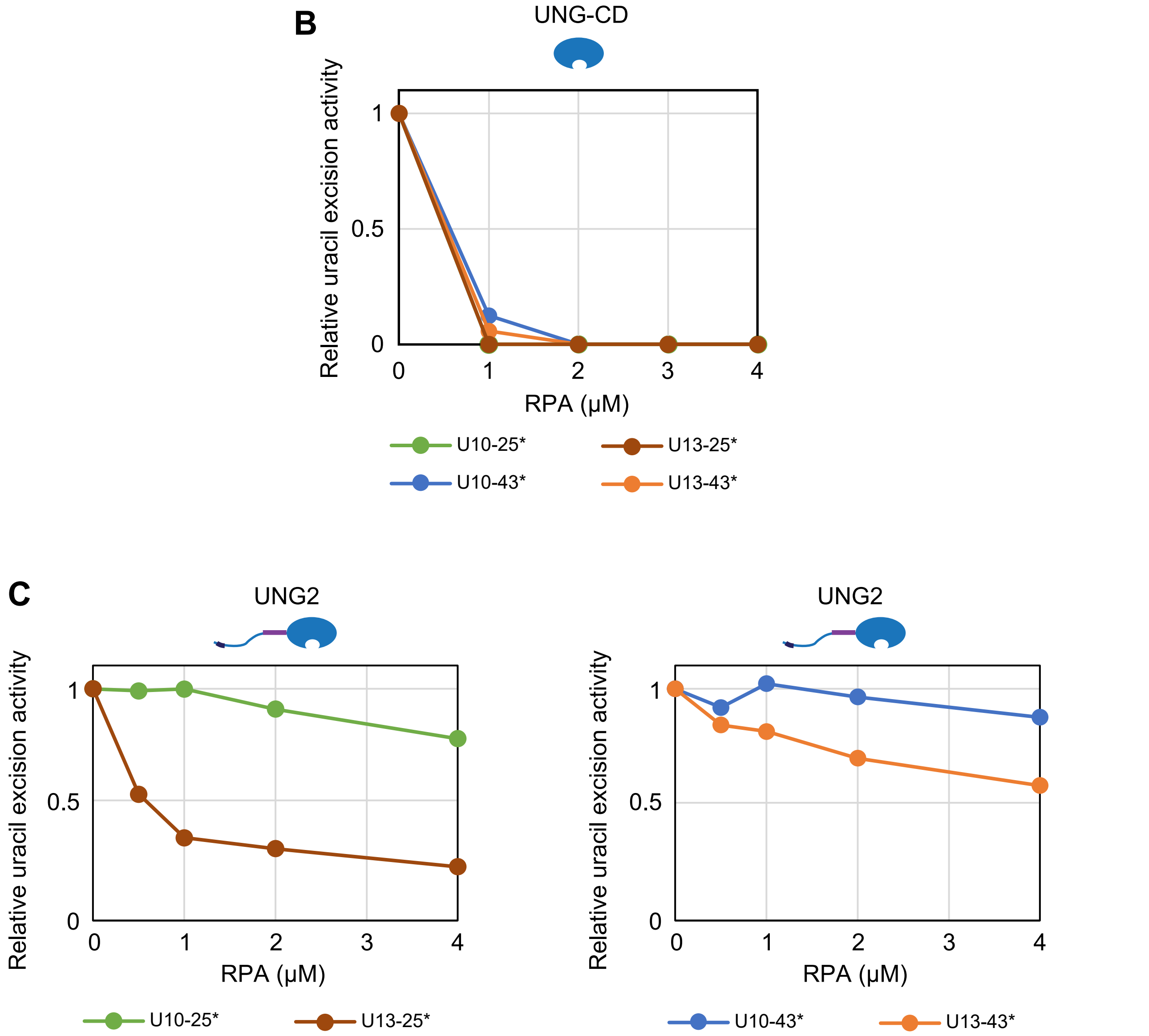

Supplementary Figure S2: Effects of uracil position and oligonucleotide length. **A)** Olignucleotide sequences. **B)** Uracil excision by the UNG catalytic domain (UNG2-CD, 0.1 nM) from the substrates (100 nM) coated with various amounts of RPA. **C)** Uracil excision by full length UNG2 (0.4 nM) from 100 nM U10-25* or U13-25* (left panel) and U10-43* or U13-43* (right panel). Note that the effect of uracil position is markedly reduced by increasing the oligonucleotide length.

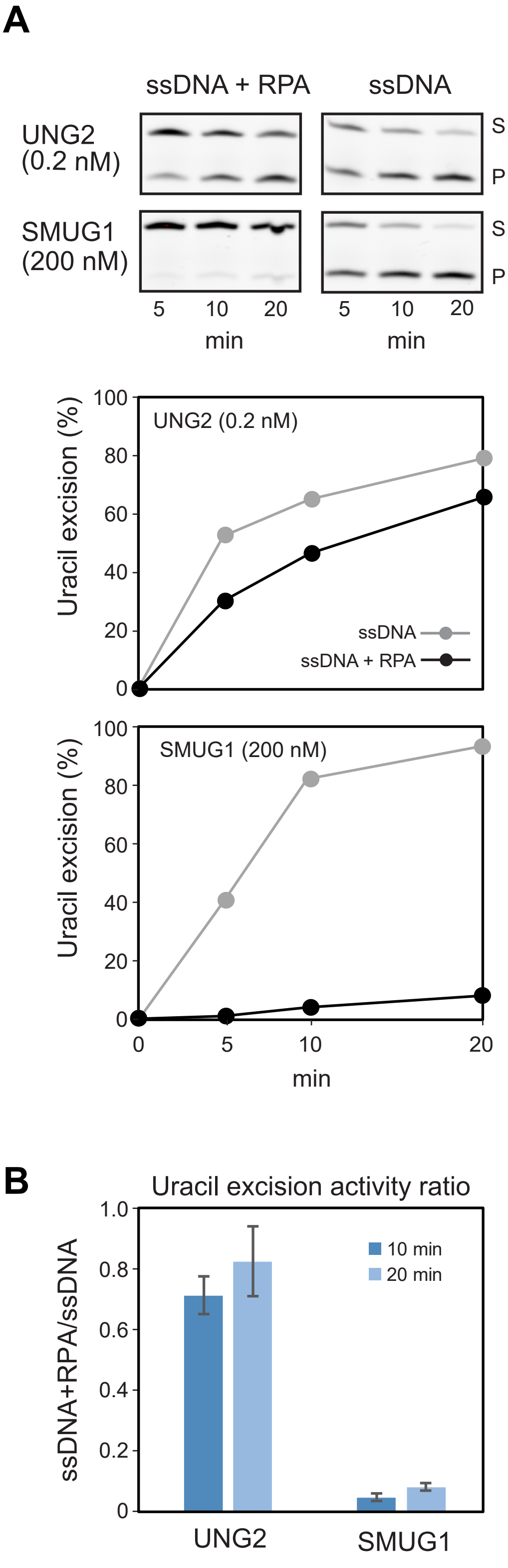

Supplementary Figure S3: Uracil in RPA-coated ssDNA is not a substrate for SMUG1.
A) Uracil excision assay comparing the ability of UNG2 and SMUG1 to target uracil in RPA-coated DNA. Activity was measured with naked and RPA (2 µM)-coated ssDNA (100 nM U13-43*). Samples were analysed after 5, 10, 20 min. Upper panel presents PAGE gels from one representative experiment. Substrate and product bands are indicated with S and P, respectively. Curves represent activity and are the calculated means from three independent experiments. **B)** Uracil-excision activity ratios for RPA-coated versus naked substrates (ssDNA+RPA/ssDNA) for UNG2 and SMUG1. Values represent the mean of three experiments with error bars as indicated.

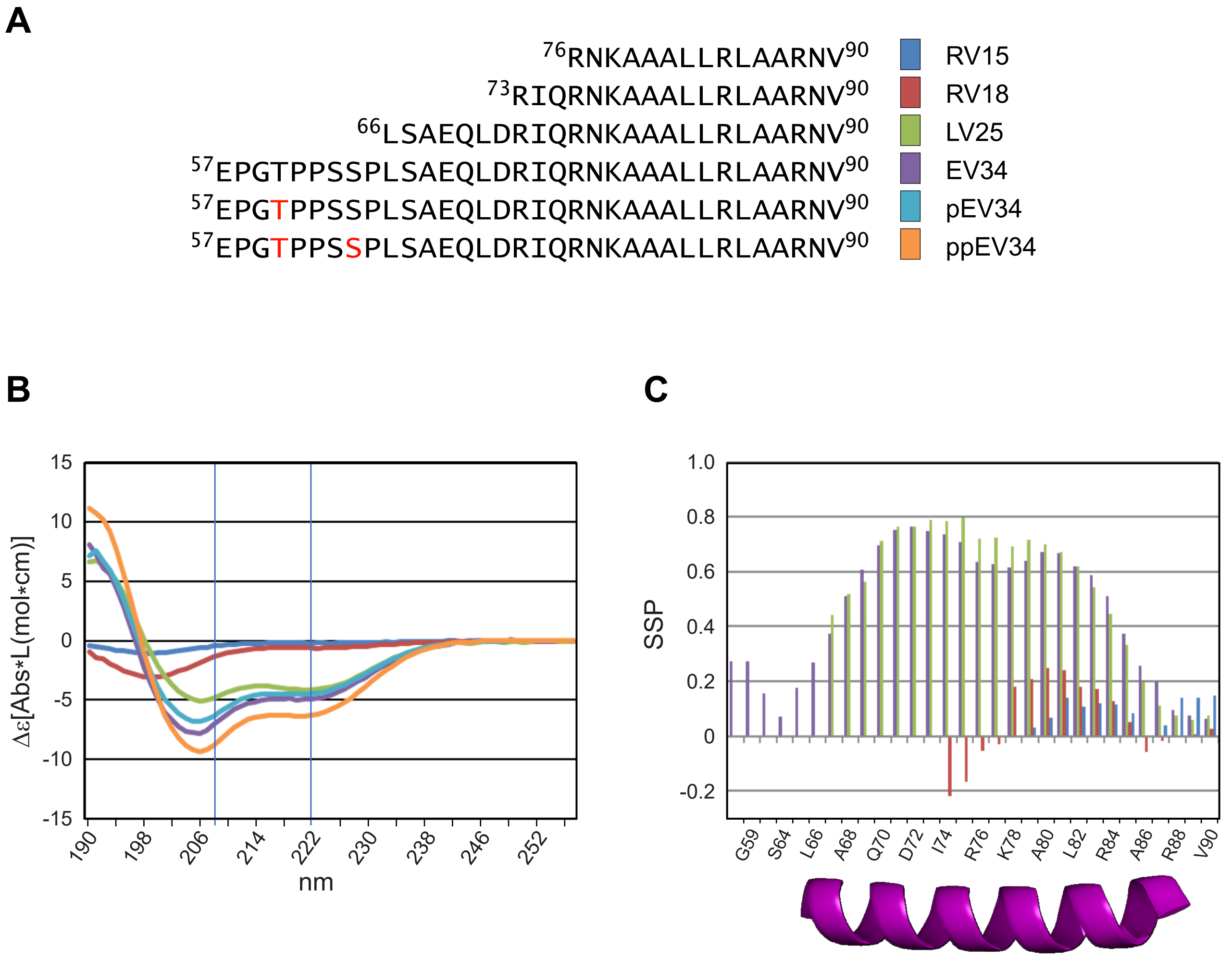

Supplementary Figure S4: Structural analysis of UNG peptides, effects of length and phosphorylation. **A)** Sequence and phosphorylation (red letters) of the different peptides. The 222/208 nm ratios indicate the helicity and are calculated from the CD spectra in B. 100% α-helix has a 222/208 nm ratio = ~0.9. All EV-34 peptides have a lower percentage of helicity than LV-25 as they have additional amino acids in random coil structure upstream of the helix. **B)** CD spectra of all six peptides show the transition from random coil structure of RV-15 and RV-18 to helical structure of LV-25. All EV-34 peptides have a helical structure similar to LV-25. Color code as in A. **C.** SSP (secondary structure propensities) prediction from NMR secondary chemical shifts shows the difference between RV-15 and RV-18 and the helical peptides LV-25 and EV-34. The helix starts at S67 and becomes less stable at the C-terminal. Color code as in A.

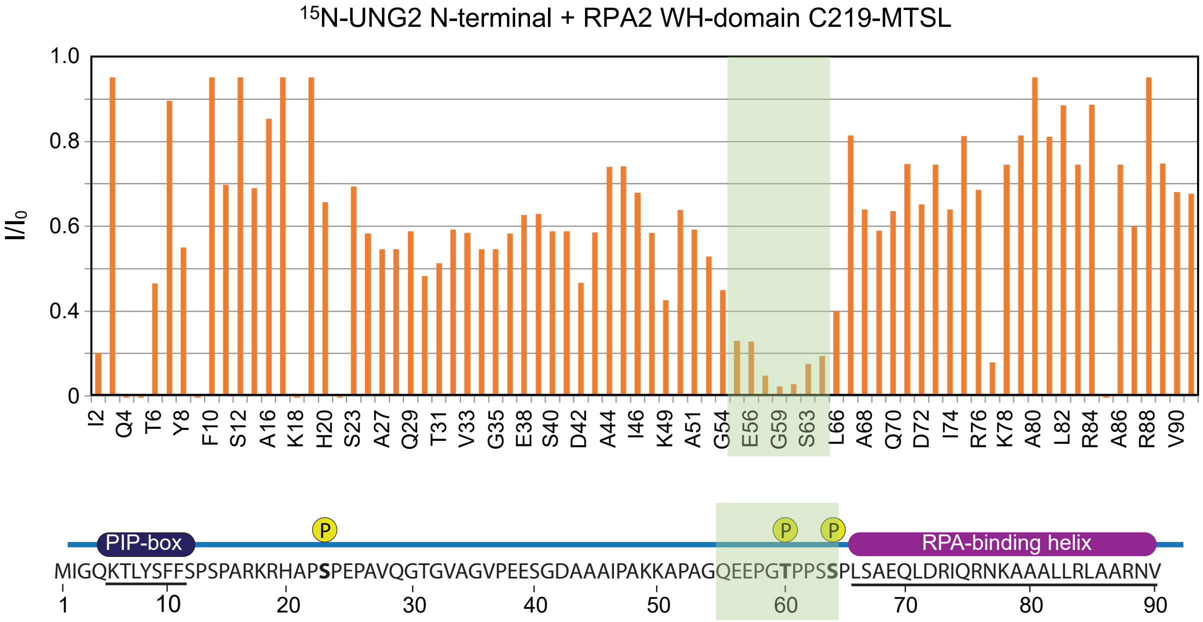

Supplementary Figure S5: NMR-based paramagnetic relaxation enhancement (PRE) measurements of UNG2 N-terminal in complex with MTSL-labelled RPA2 WH-domain.
^{15}N-labelled N-terminal region of UNG2 (N-UNG2, residues 1-93) was added in 1.5x molar excess to C219 MTSL-labelled RPA2-WH. UNG residues (Q55-S64) with reduced signal intensity due to the PRE effect of the labelled (MTSL) RPA2-WH domain are indicated (light green boxes).

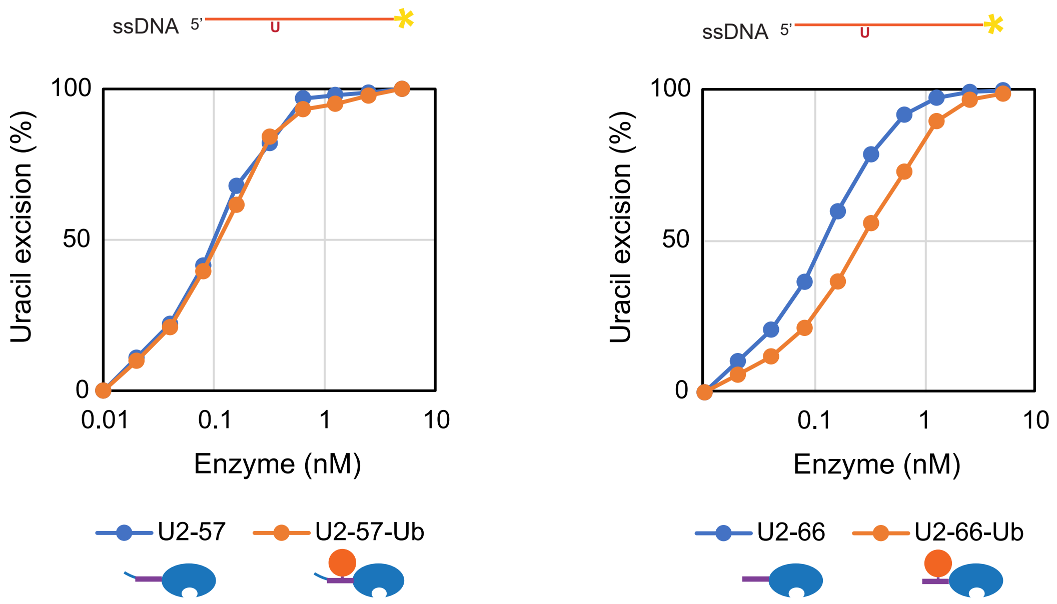

Supplementary Figure S7: Effects of K78 ubiquitinatition on naked ssDNA. U2-57 (left panel) and U2-66 (right panel) were *in vitro* ubiquitinated or mock-ubiquitinated prior to analysis against 100 nM naked ssDNA substrate. Each data point represents the mean of three (U2-57) and four (U2-66) independent experiments. The values were used together with activity data from ssDNA+RPA substrate that were run in paralell experiments (Figure 5, panels G and H) to generate the ssDNA+RPA/ssDNA activity ratios in Figure 5, panels I and J.

A

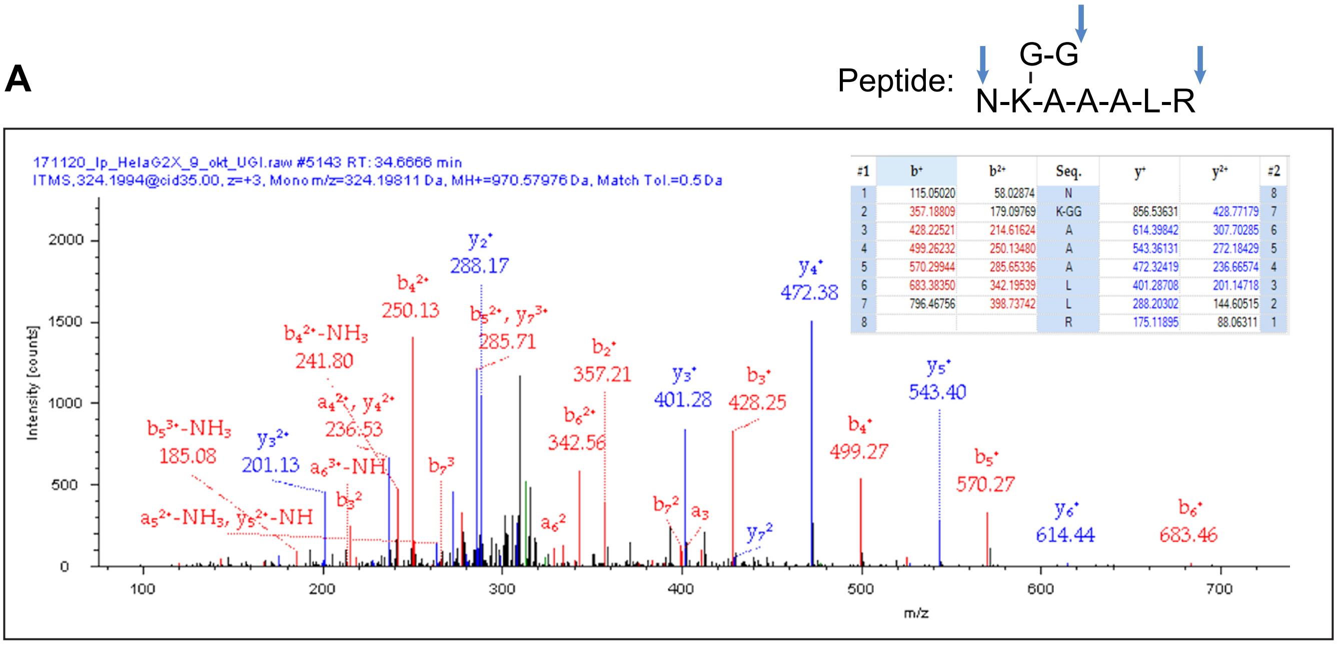

#1	b+	b2+	Seq.	y+	y2+	#2
1	115.05020	58.02874	N			8
2	357.18809	179.09769	K-GG	856.53631	428.77179	7
3	428.22521	214.61624	A	614.39842	307.70285	6
4	499.26232	250.13480	A	543.36131	272.18429	5
5	570.29944	285.65336	A	472.32419	236.66574	4
6	683.38350	342.19539	L	401.28708	201.14718	3
7	796.46756	398.73742	L	288.20302	144.60515	2
8			R	175.11895	88.06311	1

B

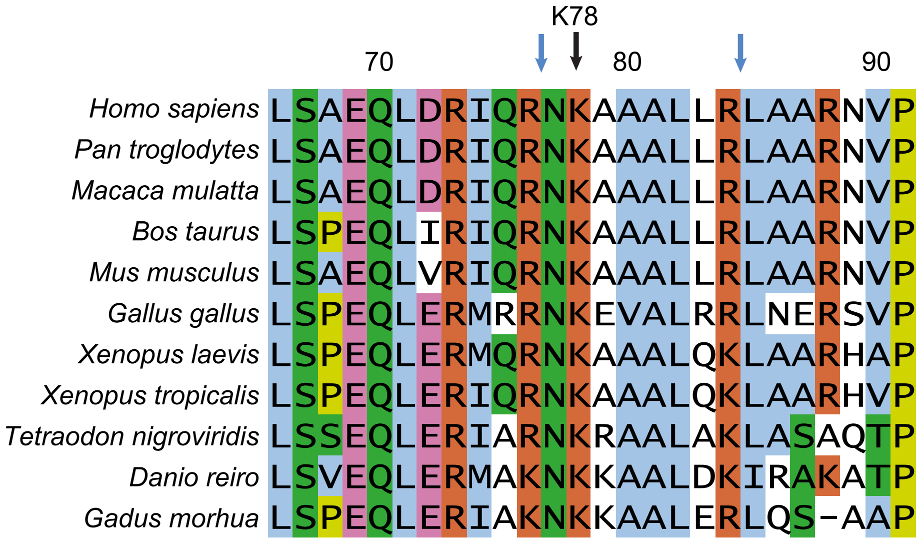

Supplementary Figure S6: UNG2 is ubiquitinated at a conserved K78 residue in the RPA-binding helix.
A) Identification of UNG2-K78 ubiquitylation (K78-Gly-Gly) in G2-enriched HeLa extract by mass spectrometry. UNG was enriched using magnetic beads coated with the UNG inhibitor Ugi and peptides generated by on-bead trypsination. A 970 Da UNG-derived peptide was identified, harbouring a GG dipeptide at K78.
B) ClustalW alignment of the N-terminal RPA-binding motif in UNG from different species. The ubiquitinylated conserved lysine and trypsin cleavage sites (in human UNG) are indicated by black and blue arrows, respectively.

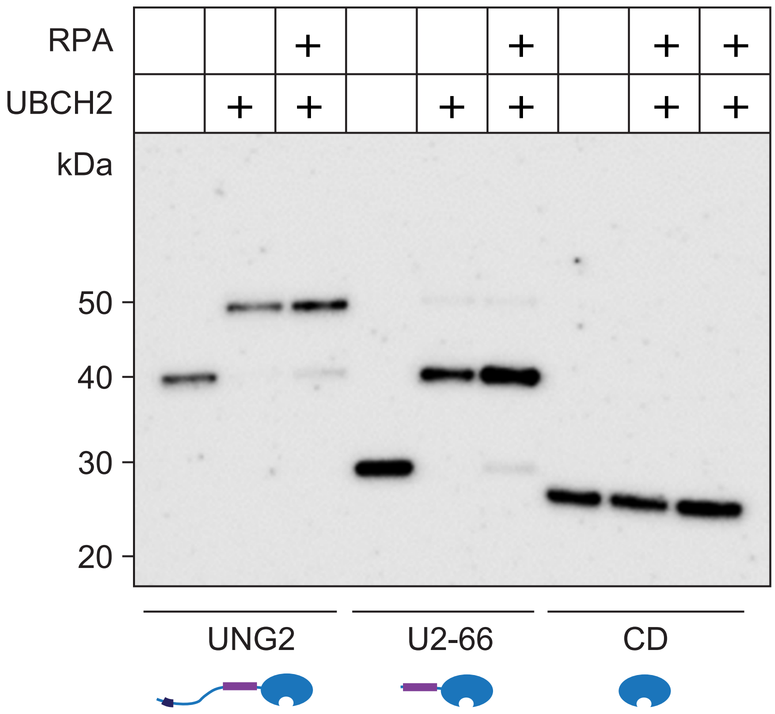

Supplementary Figure S8: UBCH2-mediated ubiquitination of K78 occurs both at unbound and RPA2-WH bound UNG2. Full-length UNG2, partially truncated U2-66 (harboring the RPA binding helix encompassing K78) and the catalytic domain (CD, lacking the RPA-binding helix) were *in vitro* ubiquitinated by UBCH2 in the presence or absence of RPA. Efficient monoubiquitiation occurred regardless of pre-binding of UNG2 and U2-66 to RPA, whereas no ubiquitination of the catalytic domain (CD) was observed.

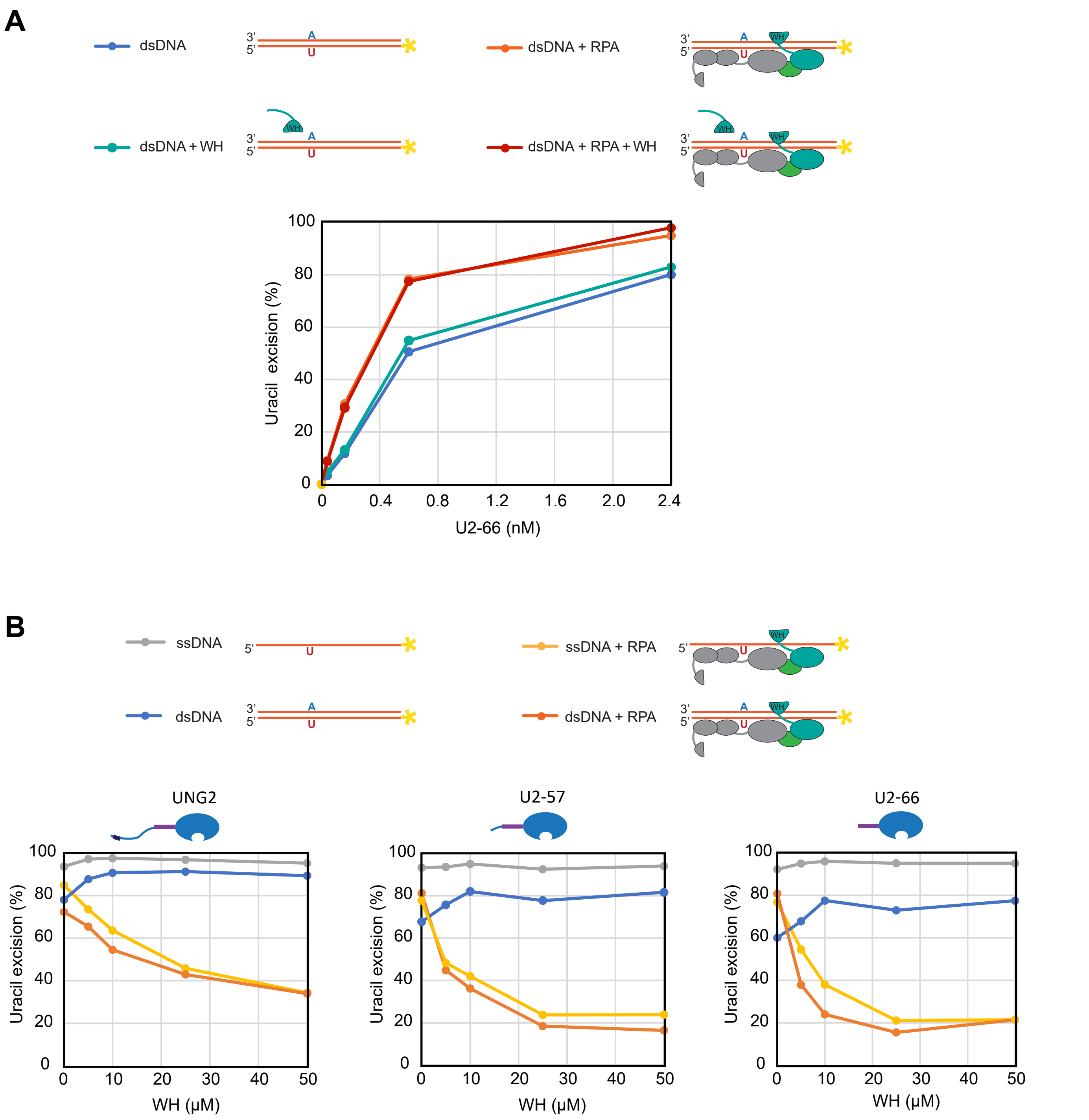

Supplementary Figure S9: Effects of purified WH-domain. A) 0-2.4 nM U2-66 was incubated with 100 nM A:U10-25* dsDNA substrate (coated or non-coated with 500 nM intact RPA) in the presence/absence of 500 nM purified WH-domain (RPA2 residues 172-270). The WH-domain did not affect uracil excision from naked or RPA-bound dsDNA, demonstrating that RPA-WH does not mediate allosteric activation of UNG. **B)** Uracil excision activities of UNG2 or N-terminally truncated versions thereof (0.4 nM) in the presence of increasing amounts (0-50 μM) of purified WH-domain. Whereas the free WH domain had little effect on uracil excision from naked ss- and dsDNA substrates, excision was markedly reduced from both RPA-bound substrates, demonstrating that the WH domain must be present as part of the RPA complex to promote uracil excision from RPA-bound substrate.

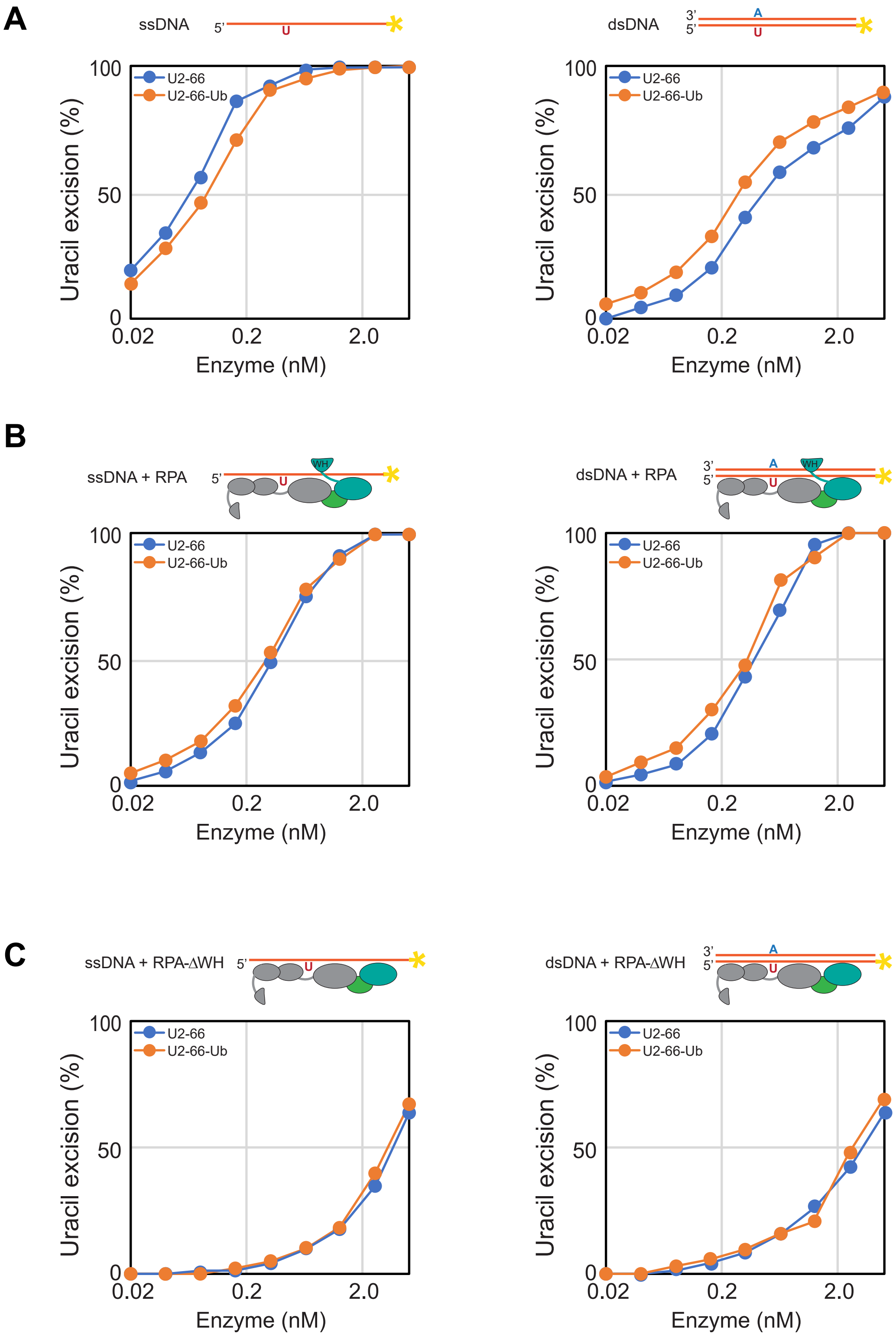

Supplementary Figure S10: Effects of K78 ubiquitination on naked versus RPA-coated ss- and dsDNA. U2-66 was *in vitro* ubiquitinated or mock-ubiquitinated prior to analysis against; **A)** naked ss- and dsDNA substrates, **B)** RPA-coated ss- and dsDNA substrates, **C)** ss- and dsDNA substrates coated with RPA-ΔWH. Reactions contained 100 nM DNA substrate and 500 nM RPA (B) or RPA-ΔWH (C). Note that in each case ssDNA and dsDNA experiments were run in parallel and by employing the same enzyme dilutions.

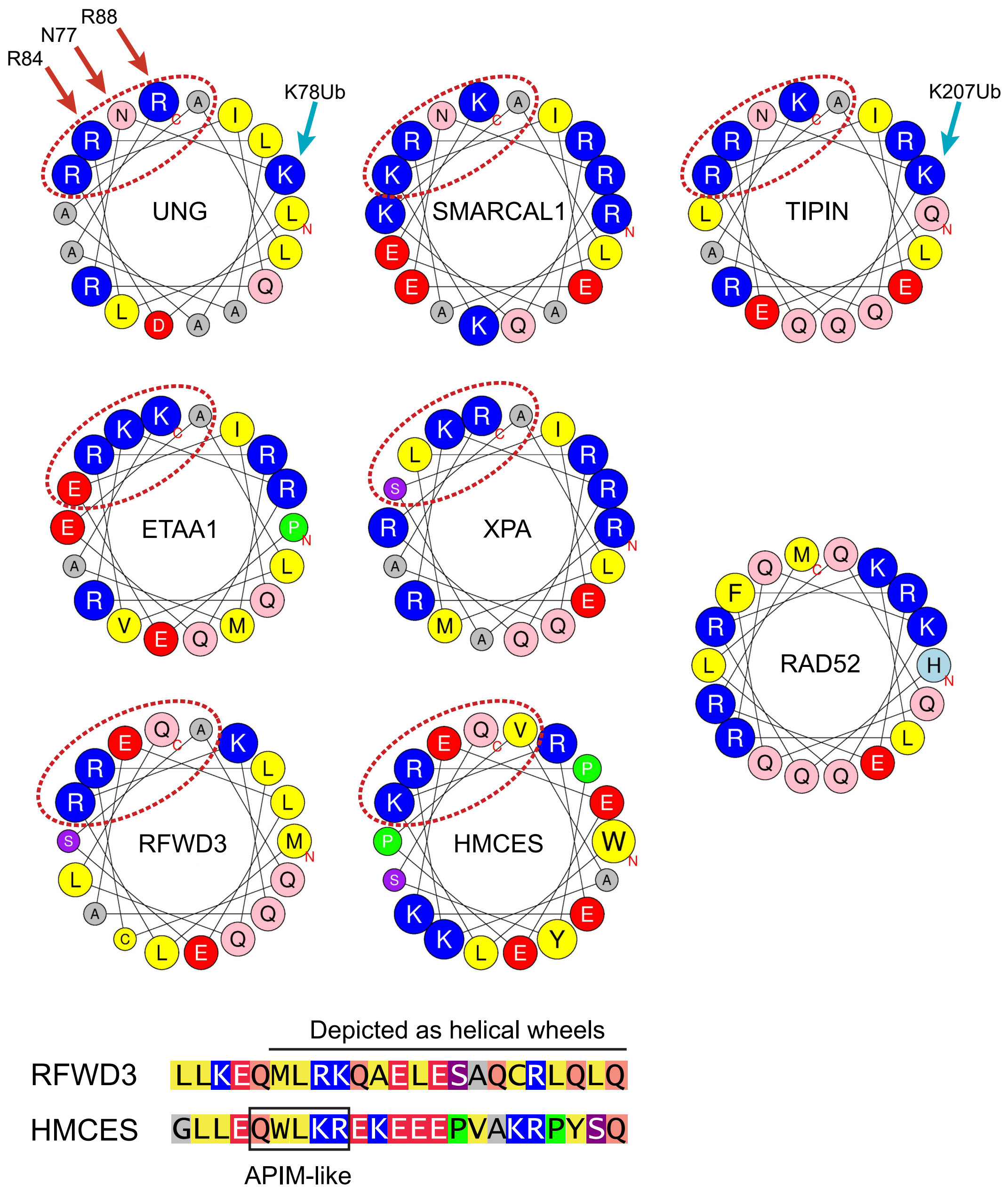

Supplementary Figure S11: Comparison of known and putative (HMCES) RPA2-WH binding motifs depicted as 18 aa helical wheels generated in HeliQuest 34 (Gautier et al., Bioinformatics 24, 2008:2101, https://heliquest.ipmc.cnrs.fr/). Residues that mediate loss of UNG activity against RPA-coated ssDNA when mutated are indicated by red arrows (residue numbering according to UNG2). The K78 ubiquitination in UNG2 and a reported K207 ubiquitination (www.phosphosite.org) in TIPIN are highlighted (blue arrows). Note the highly conserved RPA-binding sites (red circles) in UNG, SMARCAL1 and TIPIN. A slighthly different motif is present in both ETAA1 and XPA. HMCES contains a motif highly similar to that of RFWD3 and that resides in the coiled-coil domain (345-415) of RFWD3 that binds RPA2-WH (Liu et al., J. Biol. Chem. 2011:22314-22322), whereas RAD52 has a divergent motif embedded in a helix that contains elements from all groups. The putative RPA-WH-binding binding motif in HMCES overlaps with the proposed PIP-box in which W→A substitution abolished binding to PCNA (Mohni et al., Cell 176, 2019:144-153). We find that this motif rather conforms with the stress-induced PCNA-binding motif APIM (R/K- F/**W**/Y- **L**/I/V/A- L/I/V/A- K/**R**) (Gilljam et al., J. Cell Biol. 2009; 186:645–654). The putative RPA2-WH binding motif of HMCES contains two prolines, which may induce conformational flexibility to the helix (Kumeta et al., J. Cell Sci. 2018. doi: 10.1242/jcs.206326).
